# Allele-specific knockdown by an engineered DNAzyme capable of RNase H1 evasion

**DOI:** 10.1093/nar/gkaf1476

**Published:** 2026-01-06

**Authors:** Erica M Lee, Kim Nguyen, Noah A Setterholm, Turnee N Malik, John C Chaput

**Affiliations:** Department of Pharmaceutical Sciences, University of California, Irvine, CA 92697, United States; Department of Pharmaceutical Sciences, University of California, Irvine, CA 92697, United States; Department of Pharmaceutical Sciences, University of California, Irvine, CA 92697, United States; Department of Pharmaceutical Sciences, University of California, Irvine, CA 92697, United States; Department of Pharmaceutical Sciences, University of California, Irvine, CA 92697, United States; Department of Chemistry, University of California, Irvine, CA 92697, United States; Department of Molecular Biology and Biochemistry, University of California, Irvine, CA 92697, United States; Department of Chemical and Biomolecular Engineering, University of California, Irvine, CA 92697, United States

## Abstract

DNA enzymes (DNAzymes) offer an attractive therapeutic approach for targeting disease-associated mutations in mRNA transcripts, but face limitations in development due to unintended engagement by RNase H1. Although chemical optimization has led to designs with improved catalytic activity, strategies to mitigate RNase H1 recognition remain underexplored. Here, we report the incorporation of threose nucleic acid (TNA) into the backbone architecture of the 10-23 DNAzyme variant known as Dz46. Substitution of the dC3 position in the catalytic loop with TNA increases activity, whereas installation of two TNA residues in the binding arm abrogates competition by RNase H1. The resulting enzyme enables allele-specific knockdown of an oncogenic KRAS mutation in mammalian cells and facilitates general knockdown of PCSK9 and GATA3 targets. Together, these results demonstrate the utility of TNA as a chemical tool for enhancing DNAzyme performance and evading RNase H1 activity in cells.

## Graphical Abstract

**Graphical Abstract:**
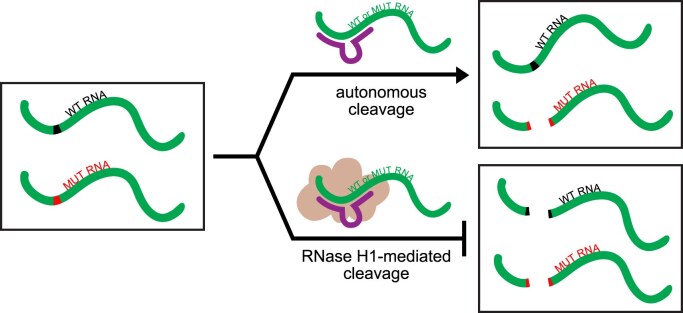

## Introduction

RNA-cleaving DNA enzymes (DNAzymes) represent a powerful class of gene silencing agents that combine the programmability of antisense oligonucleotides (ASOs) with the catalytic potential of ribozymes [1, 2]. Among the set of nucleic acid enzymes developed to date, 10-23 remains the most widely studied example due to its simple architecture, programmable binding arms, and ability to precisely catalyze site-specific cleavage at purine–pyrimidine junctions, most effectively at G–U dinucleotide junctions [3–5]. Yet despite their mechanistic appeal and favorable safety profile [6], DNAzymes have faced significant obstacles to therapeutic translation [7]. This problem is primarily due to their limited catalytic activity under physiological conditions where the concentration of free magnesium (Mg^2+^) is limiting [8] and unintended engagement by RNase H1, a cellular enzyme that utilizes the binding arms to cleave RNA following an antisense mechanism [9–11].

Allele-specific knockdown has long been a highly sought-after goal in precision medicine. In particular, there is great interest in recognizing and selectively targeting alleles that carry disease-causing single-point mutations (SNPs), which account for more than half of all disease-associated genetic mutations [12]. Although ASOs and small-interfering RNAs (siRNAs) have made significant progress toward this objective [13–15], additional work is needed to develop reagents that are generalizable and broadly applicable across the transcriptome. While protein-based gene silencing agents remain the foundation of most FDA-approved technologies [16], the intrinsic ability of DNAzymes to recognize specific dinucleotide junctions offers compelling reason for their continued development as allele-specific gene silencing agents [7].

**Figure 1.**
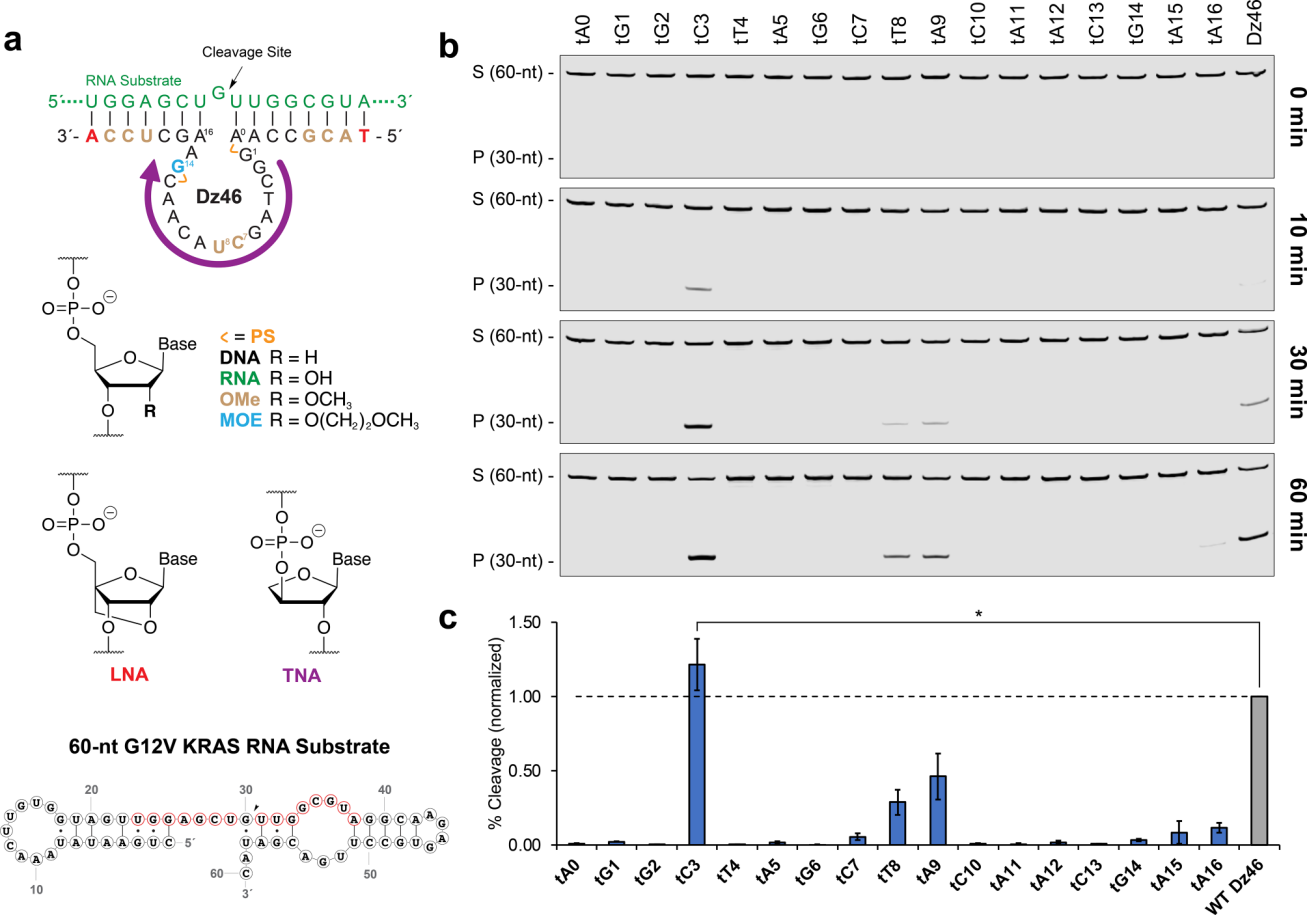
Optimizing the catalytic core of Dz46 with TNA. (**a**) Cartoon representation showing a TNA walk of the catalytic core of Dz46, chemical structures of modified nucleotides found in Dz46 analogs, and a 60-nt KRAS G12V RNA substrate depicting the region recognized by Dz46 in red used for kinetics. (**b**) Representative denaturing polyacrylamide gel electrophoresis (PAGE) gels showing 100:1 (S:E) multiple turnover RNA cleavage activity of a 60-nt substrate over the course of 60 min. (**c**) Bar graph corresponding to (b) showing RNA cleavage activity normalized to that of Dz46 at 30 min. Data presented as the mean ± standard deviation (*n* = 3 for all constructs except for Dz46 and tC3, *n* = 6 for Dz46 and tC3). Two-tailed *P*-value determined by Welch’s t-test (*, *P *< .05). All reactions were performed under simulated physiological conditions in a buffer containing 1 mM MgCl_2_, 50 mM Tris (pH 7.5), 10 mM NaCl, and 140 mM KCl at 37°C. Abbreviations: DNA (2′-deoxyribonucleic acid), RNA (ribonucleic acid), OMe (2′-deoxy-2′-methoxyribonucleic acid), MOE (2′-deoxy-2′-methoxyethoxyribonucleic acid), LNA (locked nucleic acid), TNA (threose nucleic acid), and PS (phosphorothioate). S: 5′-Cy5-labeled full-length substrate, P: 5′-Cy5-labeled cleavage product.

Chemical evolution offers a powerful approach for improving the performance of oligonucleotide therapeutics [16, 17]. Chemical modifications, particularly the incorporation of xeno-nucleic acids (XNAs) [18, 19], such as 2′-*O*-methoxy ribonucleic acid (OMe) [20], locked nucleic acid (LNA) [21], and 2′-fluoroarabino nucleic acid (FANA) [22] into the backbone structure of *in vitro* selected DNAzymes have yielded chemically modified versions of the wild-type sequence that function with enhanced catalytic activity, chemical stability, and biocompatibility [23–29]. These synthetic chemotypes have been deployed to increase nuclease stability and to enhance RNA cleavage activity [23–32]. Dz46, a chemically modified version of the classic 10-23 DNAzyme (Fig. 1a), was found to achieve ~65 turnovers in 30 min under physiological conditions, comparing favorably to the unmodified DNA sequence under forcing conditions of 50 mM MgCl_2_ (pH 8.0) [25]. However, despite a high level of chemical modification, Dz46 is still recognized by RNase H1, indicating that further modifications are needed to achieve autonomous cleavage activity in cells.

Here, we explore the use of α-l-threofuranosyl nucleic acid (TNA, Fig. 1a) as an approach for modulating DNAzyme activity. We show that TNA substitution at the dC3 position of the catalytic core of Dz46 enhances activity, while dual TNA substitutions made to the binding arm abrogate RNase H1-mediated strand cleavage. RNA and protein knockdown assays performed in mammalian cells endogenously expressing heterozygous and homozygous alleles of wild-type and mutant KRAS G12V provide strong evidence of a DNAzyme-mediated RNA cleavage mechanism that functions independent of RNase H1 engagement. Additionally, the TNA-modified Dz46 was shown to function with general knockdown activity against PCSK9 and GATA3. Together, these findings highlight the potential of TNA as a nucleic acid analog for engineering DNAzymes with improved cellular activity and provide a framework for leveraging XNA chemistry in the development of next-generation oligonucleotide therapeutics.

## Materials and methods

### General information

Oligonucleotides were synthesized in-house by solid-phase oligonucleotide synthesis on a Dr Oligo 48 DNA synthesizer (Biolytic) or purchased from Integrated DNA Technologies (Coralville, Iowa). DNA, LNA, 2′-OMe RNA, and MOE RNA phosphoramidites were purchased from Glen Research (Sterling, Virginia). TNA phosphoramidites were obtained by chemical synthesis as previously described [33]. Glen-Pak DNA Purification Cartridges were purchased from Glen Research. The Zorbax SB-C18 HPLC column (9.4 mm × 250 mm, 5 mM) for semi-preparative HPLC and Discovery C18 HPLC column (4.6 mm × 10 cm, 5 mM) for analytical HPLC were purchased from Agilent and Millipore Sigma, respectively. Full-length recombinant human RNase H1 was purchased from Abcam (Cat# Ab153634). RNase T1 was purchased from Thermo Fisher Scientific (Cat# EN0541). Dulbecco’s modified Eagle medium (DMEM) was purchased from Thermo Fisher Scientific (Cat# 10-017-CM; Waltham, MA). RPMI 1640 medium was purchased from ATCC (Cat# ATCC 30-2001). All cell lines were purchased from ATCC: NCI-H441 lung adenocarcinoma (Cat# ATCC-CRM-HTB-174), HCC827 lung adenocarcinoma (Cat# CRL-2868), SW620 colorectal adenocarcinoma (Cat# CCL-227), HeLa cervical adenocarcinoma (Cat# CCL-2),and MCF7 breast adenocarcinoma (Cat# HTB-22). Reagents used for transfection, RNA and protein-related assays purchased from different companies are listed: JetPrime DNA Transfection kit (Polyplus Transfection, France), Trizol Reagent (Invitrogen), RNA Clean & Concentrator-5 kit (Zymo Research, Cat# R1015). SuperScript III First-Strand Synthesis System (Invitrogen-Life Technologies, CA). DreamTaq DNA polymerase (Fisher Scientific, Cat# FEREP0702), Zymo DNA Clean & Concentrator-5 kit (Zymo, Cat# D4003), proteinase inhibitor cocktail Set VII (EMB, Cat# 539138-1ML), Turbo DNase (Life Tech., Cat# AM2239), RNase A (Thermo Fisher, Cat# FEREN0531), BCA assay (BioSciences, Cat# 786-570), and 4%–20% gradient 10-well Mini-PROTEAN gel (Bio-Rad, Cat# 4561094). Trans-Blot Turbo Transfer unit (Bio-Rad, Cat# 1704150), Intercept (TBS) Protein-Free Blocking buffer (IBB) (Licor, Cat# LIC-927-80003). Antibodies were purchased from Proteintech and Santa Cruz Biotech: rabbit anti-KRas (Proteintech, Cat# 12063-1-AP), mouse anti-beta-Actin (Proteintech, Cat# 66009-1-1g), mouse monoclonal Gapdh antibody (Proteintech, Cat# 60004-1-IG), mouse monoclonal Pcsk9 antibody (Santa Cruz Biotech, Cat#: sc-515082), and rabbit polyclonal Gata3 antibody (Proteintech, Cat# 10417-1-AP). Secondary antibodies were ordered from Licor: Licor IRDye^®^ 800CW Anti-Rabbit IgG Goat Secondary Antibody (Licor, Cat# 926-32211), Licor IRDye^®^ 680RD Anti-Mouse IgG Goat Polyclonal Goat Secondary Antibody (Licor, Cat# 926-68 070).

### DNAzyme synthesis

All DNAzymes were prepared by solid-phase oligonucleotide synthesis using an automated Dr Oligo DNA synthesizer on a 1 μmol scale universal CPG column (Glen Research) with optimized coupling times for modified phosphoramidites. Oligonucleotides were removed from the column and deprotected by incubation in ammonia gas for 2 h at 80°C (60 psi), and purified on a Glen-Pak™ DNA Purification Cartridge (Glen Research) followed by ion-pair reversed-phase HPLC as previously described [34]. The samples were lyophilized to dryness, resuspended in water, and UV quantified. The synthesized DNAzymes were sodium salt exchanged prior to use in cells and verified by MALDI-TOF mass spectroscopy.

### Cleavage product analysis

To generate an RNA ladder, 1 μM of Cy5-labeled 60-nt RNA was digested by 1 U of RNase T1 (1000 U/μl) in 50 mM Tris (pH 7.5), 10 mM NaCl, and 140 mM KCl at room temperature for 1 min. For DNAzyme-catalyzed reactions, 250 nM Dz and 250 nM Cy5-labeled 60-nt RNA (1S:1E) were incubated in the same buffer used for RNase T1 digestion but with the addition of 1 mM MgCl_2_. For both reactions, a 1.5 μl aliquot of the reaction mixture was removed and quenched in 16.5 μl formamide buffer [99% deionized formamide, 25 mM ethylenediaminetetraacetic acid (EDTA)]. Quenched samples were denatured at 95°C for 10 min and resolved using 15% denaturing PAGE. Gels were imaged using an Odyssey CLx imaging system (LI-COR) and quantified using Image Studio Lite (LI-COR).

### TNA walk of the catalytic domain

DNAzyme variants were evaluated for activity under multiple turnover conditions with 10 nM Dz and 1000 nM 16-nt RNA substrate (100S:1E) in simulated physiological reaction buffer comprised of 50 mM Tris–HCl (pH 7.5), 10 mM NaCl, 140 mM KCl, and 1 mM MgCl_2_. Dzs and substrates were annealed in reaction buffer without MgCl_2_ by heating for 5 min at 95°C and then cooling for 5 min at 4°C. Reactions were equilibrated to 37°C for 2 min before initiating the reaction with the addition of MgCl_2_. After heating at 37°C for 30 min, a 1.5 μl aliquot of the reaction mixture was removed and quenched in 16.5 μl formamide stop buffer (99% deionized formamide, 25 mM EDTA). Quenched samples were denatured at 95°C for 10 min and resolved using 20% denaturing PAGE. Gels were imaged using an Odyssey CLx imaging system (LI-COR) and quantified using Image Studio Lite (LI-COR).

### Kinetic assay

Dz46 variants were assayed under multiple turnover conditions with 50 nM Dz and 500 nM (10S:1E) and 10 nM Dz and 1000 nM (100S:1E) 60-nt RNA substrate in simulated physiological reaction buffer comprised of 50 mM Tris–HCl (pH 7.5), 10 mM NaCl, 140 mM KCl, and 1 mM MgCl_2_. Reactions were equilibrated to 37°C for 2 min (no annealing step) before initiating the reaction with the addition of MgCl_2_ and kept at 37°C throughout the time course. At designated times between 0 and 60 min, 1.5 μl aliquots of the reaction mixture were removed and quenched in 16.5 μl formamide stop buffer (99% deionized formamide, 25 mM EDTA). Quenched samples were treated and analyzed as described earlier.

### Initial velocity (v_0_) determination

Initial rates (v_0_) were calculated by a linear fit of the first 10%–15% RNA cleavage events observed in the kinetic assay using Equation (1):


(1) \begin{eqnarray*}
{{\mathrm{ v}}_o} = \frac{{\left( {{{\mathrm{ \mathit{ Y}}}_f} - {{\mathrm{ \mathit{ Y}}}_o}} \right) \times \left[ {{{\mathrm{ \mathit{ S}}}_0}} \right]}}{{\left( {{{t}_f} - {{t}_0}} \right)}},
\end{eqnarray*}

where v_0_ is the initial velocity (nM*min^−1^), *Y*_f_ is the percentage of cleaved substrate at finite time *t* when the first 10%–15% cleavage is reached, *Y*_0_ is the percentage of cleaved substrate at *t*_0_, [*S*]_0_ is the initial substrate concentration (nM) at t_0_.

The percentage of cleaved substrate (*y*-axis) and reaction time (*x*-axis) in minutes (min) was fitted to the pseudo-first-order association kinetics of a substrate and an enzyme Equation (2) using Prism 9 (GraphPad, USA) to graph the kinetic curve:


(2) \begin{eqnarray*}
Y = {{Y}_0} + \left( {{{Y}_\infty } - {{Y}_0}} \right) \times \left( {1 - {{e}^{ - {{k}_{obs}}{\mathrm{\ }} \times {\mathrm{\ }}t}}} \right),
\end{eqnarray*}

where *Y* is the percentage of cleaved substrate at finite time *t, Y*_0_ is the percentage of cleaved substrate at t_0_, *Y*_∞_ is the percentage of cleaved substrate at infinite time where the reaction is plateau, and *k*_obs_ is the observed pseudo-first-order (substrate/enzyme interaction) rate constant (min^−1^).

### RNase H1 analysis

To evaluate RNase H1 recognition of the Dz-substrate complexes, cleavage assays were performed in the presence and absence of RNase H1. Cleavage activity was evaluated using 250 nM of Dz or ASO mixed with the 60-nt RNA substrates in a 1:1 ratio: 125 nM WT KRAS with a 5′-Cy5 tag and 125 nM G12V KRAS with a 5′-Alexa Fluor 750 tag. Reactions were performed at 37°C in simulated physiological reaction buffer containing 50 mM Tris–HCl (pH 7.5), 10 mM NaCl, 140 mM KCl, and 1 mM MgCl_2_, with or without 5 ng/μl human RNase H1. Aliquots (1.5 μl) were removed after 30 min, quenched, and resolved using 20% denaturing PAGE as described earlier.

### Melting temperature (Tm) study

All melts were performed with 1:1 oligonucleotide stoichiometry (1 μM DNAzyme + 1 μM RNA substrate) in 1 M NaCl, 50 mM Tris–HCl, 1 mM MgCl_2_ at pH 7.5. Prior to melting, the strands are annealed in an Eppendorf tube by heating to 95°C for 5 min and cooling on ice. Melting curves were obtained in the reverse and forward melting directions in a quartz cuvette of 1-cm path length with a temperature gradient of 20° to 90°C and a ramping rate of 1°C per min by monitoring the change in UV absorbance at 260 nm across the temperature range using a Cary 100 UV-Vis spectrophotometer equipped with a Peltier thermal controller. Tm values were determined by the hyperchromicity method using the Cary software package.

### Cell lines and mammalian cell culture conditions

MCF7, HCC827, SW620, and NCI-H441 cells were cultured in RPMI 1640. HeLa was cultured in DMEM. Media were supplemented with 10% fetal bovine serum and 1% penicillin-streptomycin (1 mg/ml) and grown at 37°C, 5% CO_2_.

### Transfection

Cells were seeded in 6-cm (5 ml) culture dishes and allowed to grow for 24–48 h until they reached 40%–50% confluency. Cells were treated with 500 nM Dz using the JetPrime DNA Transfection Kit according to manufacturer’s instructions with the exception that 40 μl of JetPrime reagent and 250 μl of JetPrime buffer were used per transfection of a 5 ml culture. The 500 nM Dz treatment was administered as a double-dosing regimen with the first transfection of 250 nM Dz at 0 h and a second transfection of 250 nM Dz at 6 h for HeLa cells and 24 h for NCI-H441, HCC827, SW620, and MCF7 cells. HeLa cells were harvested at 24 h and NCI-H441, HCC827, SW620, and MCF7 cells were harvested at 48 h.

### Total RNA isolation and reverse transcription polymerase chain reaction

Total RNA isolation was performed on each cell pellet using 1 ml/pellet of Trizol Reagent according to the manufacturer’s instructions. The extracted total RNA was resuspended in 44 μl of water. Total RNA was then treated with Turbo DNAse (20 U/reaction) at 37°C for 30 min with shaking, followed by purification using the RNA Clean & Concentration-5 Kit according to manufacturer’s recommended protocol. Total RNA was eluted from IC columns twice with 22 μl of water, resulting in a total volume of 44 μl. Two hundred to one thousand nanograms of DNA-free RNA was subjected to complementary DNA (cDNA) synthesis using the SuperScript III First-Strand Synthesis System with random hexamer primers in a 20-μl reaction according to the manufacturer’s instructions. Template messenger RNA (mRNA) in the RNA:cDNA hybrid was removed by RNase H (2U/cDNA reaction) digestion, resulting in a final volume of 21 μl. cDNA was then diluted to a final concentration of 5 ng/μl based on the starting amount of total RNA used in the cDNA synthesis and taken forward to PCR. Per 25-μl PCR reaction, 5 to 10 μl of 5 ng/μl cDNA was used. PCR thermal parameters used: 1× (94°C, 2 min), 40× (94°C, 20 s; 57°C, 15 s; 70°C, 10 s), 1× (70°C, 5 min) for HCC827, HeLa, and MCF7 cells; SW620 cells used 18× amplification. Refer to Supplementary Table S8 for primers used in this assay.

### Restriction fragment length polymorphism assay

PCR-restriction fragment length polymorphism assay PCR-RFLPA was used to differentiate WT and G12V KRAS alleles as previously described [25, 35, 36]. In brief, cDNA was amplified by PCR using a unique sense primer that carries a single substitution (G to C) at the first nucleotide position (bold) of KRAS codon 11 (GCT to CCT). This substitution introduces *BstN*I recognition site (“CCWGG”), which spans from KRAS codon 11 (CCT) to codon 12 (GGT), in WT KRAS but not the G12V allele. Treatment of the amplicons with *BstN*I endonuclease shortens the WT allele by 50 nts relative to the G12V allele, making the two alleles distinguishable by 3% agarose gel electrophoresis. Refer to Supplementary Table S9 for primers used in this assay.

### Total protein isolation and immunoblot analysis

At 24 and 48 h post-transfection, cells were scraped from the plates and spun down at 1000 × *g* for 5 min at room temperature. Cell pellets subjected to protein isolation were resuspended with 50–150 μl (10× volume of dried cell pellet) of RIPA lysis buffer [50 mM Tris–HCl at pH 7.5, 150 mM NaCl, 0.1% Triton, 0.5% Na-deoxycholate & 0.1% sodium dodecyl sulfate (SDS), 5 mM EDTA]. Eight microliters of Protease Inhibitor Cocktail Set VII, 4 μl of Turbo DNase, and 4 μl of RNase A were supplemented per 1 ml of RIPA Lysis buffer. Resuspended cells were incubated on ice for 0.5 h, subjected to three freeze-thaw cycles to lyse cells, and centrifuged at 15 000 rpm for 20 min at 4°C to collect the supernatant containing total protein. Total protein lysate was subjected to a BCA assay at 37°C for 30 min and quantified using the NanoDrop BCA application to determine protein concentration. Fifteen to thirty micrograms of total protein was resolved in a 4%–20% gradient 10-well Mini-PROTEAN gel. The resolved proteins were transferred to a nitrocellulose membrane using a Trans-Blot Turbo Transfer unit. The membrane was allowed to air-dry for 0.5 h and blocked with Intercept Protein-Free Blocking Buffer (IBB) for 1 h at RT. The membrane was probed overnight at 4°C with fresh IBB supplemented with 0.2% Tween-20 (IBBT) + rabbit anti-Kras at 1:2000 dilution or mouse anti-β-actin (loading control) at 1:10 000 dilution, and then for an additional 1 h at RT on the following day. For Pcsk9, mouse monoclonal antibody (1:500 dilution) was used. For Gata3, rabbit polyclonal antibody (1:1000 dilution) was used. Mouse monoclonal Gapdh antibody (1:10 000 dilution) was used for the loading control for Pcsk9 and Gata3 blots. Blots were washed 3 × 10 min each with 1× TBST (150 mM NaCl, 50 mM Tris–HCl, pH 7.6 + 0.1% Tween-20) at RT and subsequently incubated in 0.02% SDS-supplemented-IBBT + Licor IRDye^®^ 800CW Anti-Rabbit IgG Goat Secondary Antibody or Licor IRDye^®^ 680RD Anti-Mouse IgG Goat Polyclonal Goat Secondary Antibody at 1:20 000 dilution at RT for 1 h. Finally, blots were washed 3 × 10 min each with 1× TBST at RT. Blots were imaged using an Odyssey CLx imaging system (LI-COR) and quantified using Image Studio Lite (LI-COR).

## Results

We set out to optimize Dz46 through an iterative chemical evolution strategy involving rational design, synthesis, and testing of chemically modified variants [25]. Our goal was to identify positions within the DNAzyme where substitutions with XNAs, specifically TNA, could enhance catalytic activity and evade RNase H1 recognition. TNA, an RNA analog with a four-carbon threose sugar replacing the five-carbon ribose sugar found in RNA, was selected for its nuclease resistance and ability to hybridize with DNA and RNA [37, 38]. Since TNA amidites and oligonucleotides are not widely available from commercial sources, the TNA-modified Dz46 variants were prepared by solid-phase oligonucleotide synthesis (Supplementary Tables S1–S6) using chemically synthesized TNA phosphoramidites, and the resulting oligonucleotides were purified by ion-pair reversed-phase high-performance liquid chromatography (HPLC) [33, 34]. Natural DNA and RNA oligonucleotides were obtained from commercial sources (Supplementary Tables S7–S9).

To ensure physiological relevance, all DNAzyme activity assays were performed under multiple turnover conditions in buffer mimicking the ionic strength of the intracellular environment [1 mM MgCl_2_, 140 mM KCl, 10 mM NaCl, and 50 mM Tris–HCl (pH 7.5)] at 37°C. The functional impact of each chemical perturbation toward DNAzyme activity was analyzed by denaturing PAGE, and initial velocities (v_0_) of the final constructs selected for analysis were determined using a 60-nucleotide (nt) RNA substrate at a 100:1 substrate-to-enzyme ratio (S:E). The DNAzyme cut site of the 60-nt substrate was confirmed by an RNA ladder generated by RNase T1, an endonuclease that cleaves single-stranded RNA after guanosine residues, producing a series of truncations, including the expected 30-nt product (Supplementary Fig. S1).

### Catalytic core optimization

We first performed a TNA walk of the catalytic loop (positions 1–15 and flanking sites 0 and 16) to assess the tolerance of each position to chemical perturbation (Fig. 1a). Although this region of the sequence had previously undergone extensive optimization, its compatibility with TNA remained unexplored [25]. We generated 17 TNA-substituted Dz46 variants, each retaining the complete set of Dz46 modifications, including LNA and OMe in the binding arms, and 2′-*O*-methoxyethoxy ribonucleic acid (MOE), OMe, and phosphorothioate (PS) in the catalytic loop (Fig. 1a). The catalytic activity of each variant was initially evaluated using a short size-matched 16-nt RNA substrate under 100:1 (S:E) multiple turnover conditions (Supplementary Fig. S2). The resulting cleavage profile reveals that substitutions made to positions 3, 8, and 9 enhance catalytic activity, while positions 0, 2, 4, 6, and 10 were intolerant to the TNA modification (Supplementary Fig. S2). The tolerance of position 8 toward substitution with sugar-modified analogs [25–28] aligns with its placement near metal-ion binding site II, as reported previously for the NMR structure of 10-23 [39]. However, the tolerance of TNA substitution at positions 3 and 9 was thought to be more interesting, as these residues, to the best of our knowledge, were not previously identified as permissive sites of chemical modification, making them novel sites for chemical optimization.

To examine the robustness of the substitutions, the TNA walk was repeated using a 60-nt RNA substrate under 10:1 and 100:1 (S:E) multiple turnover conditions (Fig. 1b and c and Supplementary Fig. S2). Several observations were made by comparing the results from the assays performed using different substrate lengths and concentrations. First, DNAzyme activity observed on the 60-nt substrate mirrors the activity observed for the 16-nt substrate. Second, DNAzyme activity is reduced under the more challenging conditions of increased substrate concentration. Third, the difference in activity between variants with TNA substitutions at positions 3 and 9 becomes more evident under the stringent conditions (Fig. 1b and c), emphasizing the enhanced activity of the variant with TNA at position 3. This last observation is consistent with a noticeably slower bursting profile for the variant with TNA at position 9 as compared to the variant with TNA at position 3 (Supplementary Fig. S3). In a 60-min time course under 100:1 (S:E) conditions on the 60-nt substrate, the variant with TNA at position 3 reaches 50% RNA cleavage at ∼30 min and maintains an enhanced catalytic activity relative to that of the original Dz46 (Fig. 1b and c) [25].

### Binding arm optimization

Next, we investigated whether TNA substitutions in the binding arms could reduce RNase H1 recognition without compromising catalytic function. Previous studies have implicated RNase H1 as the primary driver of targeted RNA cleavage in cultured mammalian cells treated with DNAzymes [9–11]. In such cases, competition is thought to exist between the intrinsic activity of the DNAzyme and RNase H1, which recognizes complementary DNA–RNA duplexes as substrates for cleavage [40]. Studies have shown that RNase H1 requires a minimum of four consecutive base pairs (bp) to trigger cleavage of the RNA [40, 41, 42], but it is noted that the DNA “gap” requirement may deviate by 1 to 2 additional bp due to the helical structure of the duplex with various positions and modifications considered [43]. Dz46, even with its current modifications at the terminal positions of the binding arms, still meets the minimum 4 bp requirement and contains segments in the catalytic core region where off-target binding could, in theory, occur. Ultimately, however, the balance between the two mechanisms, DNAzyme- versus RNase H-mediated strand cleavage, depends on the type and extent of chemical modifications found in the DNAzyme, with unmodified sequences driven by RNase H1 [9–11] and modified sequences favoring a DNAzyme-mediated cleavage mechanism [25].

One model for evaluating RNase H1 recognition of the DNAzyme-RNA complex involves a two-color analysis of allele-specific RNA cleavage [25]. In this assay, synthetic RNA strands for wild-type and mutant substrates are separately prepared with 5′-modified Cy5 (red) and Alexa Fluor 750 (green) tags, respectively. In our case, we use an RNA sequence corresponding to KRAS G12V, a prominent mutation found in many human cancers [44]. Since DNAzymes can be programmed to exclusively cleave only one of the alleles, assays performed in the presence of both allelic substrates should exclusively yield a cleavage product for the on-target KRAS G12V strand. However, because the DNAzyme binds, but does not cleave, the off-target wild-type strand, assays performed in the presence of RNase H1 can yield one or more alternative-length products due to RNase H1-mediated cleavage of the off-target strand. Although RNase H1-mediated cleavage of the on-target strand is possible, this reaction is rarely observed as the DNAzyme can kinetically outcompete RNase H1 for the correct allelic target. For example, Dz46 has been shown to yield a longer-length product due to unwanted RNase H1-mediated cleavage of the wild-type KRAS strand with no interference of the KRAS G12V strand [25]. One minor caveat of this assay is that Alexa Fluor 750 is prone to signal bleed from the 800 nm (green) channel to the 700 nm (red) channel in the infrared Odyssey CLx imaging system, which can be excluded from analysis by separately evaluating each substrate (Supplementary Fig. S4).

**Figure 2.**
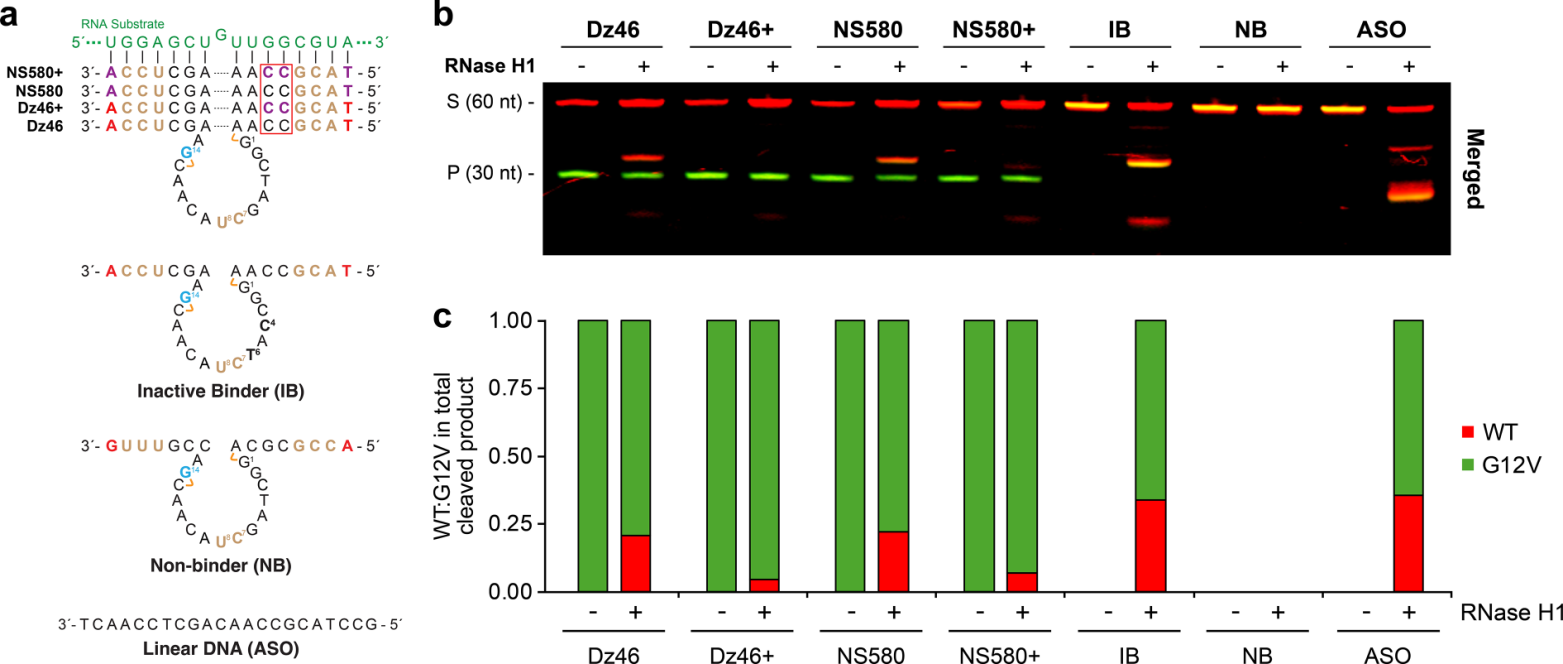
Optimizing the binding arms of Dz46 for reduced RNase H activity. (**a**) Variants of Dz46 carrying TNA in the binding arm, along with the non-binder (NB), inactive binder (IB), and ASO controls. (**b**) Representative denaturing PAGE gel showing RNA cleavage profiles in reactions containing both the wild-type (red) and G12V (green) substrates (1:1) in the absence (−) or presence (+) of 5 ng/μl human RNase H1 after a 30 min incubation (*n* = 2). Merged images of Cy5 (red) and AlexaFluor750 (green) channels are shown. (**c**) Bar graph corresponding to (b) showing the fraction of G12V and WT cleavage in the absence and presence of RNase H1. Reactions were performed under simulated physiological conditions in a buffer containing 1 mM MgCl_2_, 50 mM Tris (pH 7.5), 10 mM NaCl, and 140 mM KCl at 37°C. S: 5′-labeled full-length substrate, P: 5′-labeled cleavage product. DNAzyme chemistry is color-matched to Fig. 1.

The two-color RNase H1 assay was validated with standard controls that include inactive (IB) and non-binding (NB) versions of Dz46 as well as an ASO, which behave as expected. The inactive DNAzyme and ASO controls yield cleavage products only in the presence of RNase H1, while the non-binding DNAzyme shows no signs of RNA cleavage, regardless of whether RNase H1 is present or absent (Fig. 2). For the IB control, preferential cleavage of the G12V substrate by RNase H1 was observed (Fig. 2b and c). This is likely due to increased hybridization of the DNAzyme to the on-target G12V substrate versus the off-target wild-type substrate, which carries a 1 bp mismatch in the binding arm. Together, the controls provide evidence of an existing antisense mechanism that competes with the intrinsic catalytic activity of the DNAzyme.

In an effort to reduce RNase H1 recognition of the enzyme-substrate complex, we examined Dz46 and an analog of Dz46 (NS580) that contain and lack TNA in their binding arms (Fig. 2a). In the absence of RNase H1, all four DNAzymes (Dz46, Dz46+, NS580, and NS580+) exclusively cleave the mutant substrate, demonstrating >99% specificity for the target allele (Fig. 2). However, in the presence of RNase H1, Dz46- and NS580-treated samples yield the desired G12V cleavage product as well as a longer-length RNase H1-mediated product caused by cleavage of the off-target wild-type allelic strand (Fig. 2 and Supplementary Fig. S5). Strikingly, the RNase H1-mediated product is largely abrogated in the samples treated with Dz46+ and NS580+, which carry TNA in their binding arms. The reduced ability for RNase H1 to recognize DNAzymes carrying TNA modifications in their binding arms favors an autonomous DNAzyme-mediated cleavage mechanism that functions independent of any cellular protein machinery.

### Combining catalytic and binding arm modifications

**Figure 3.**
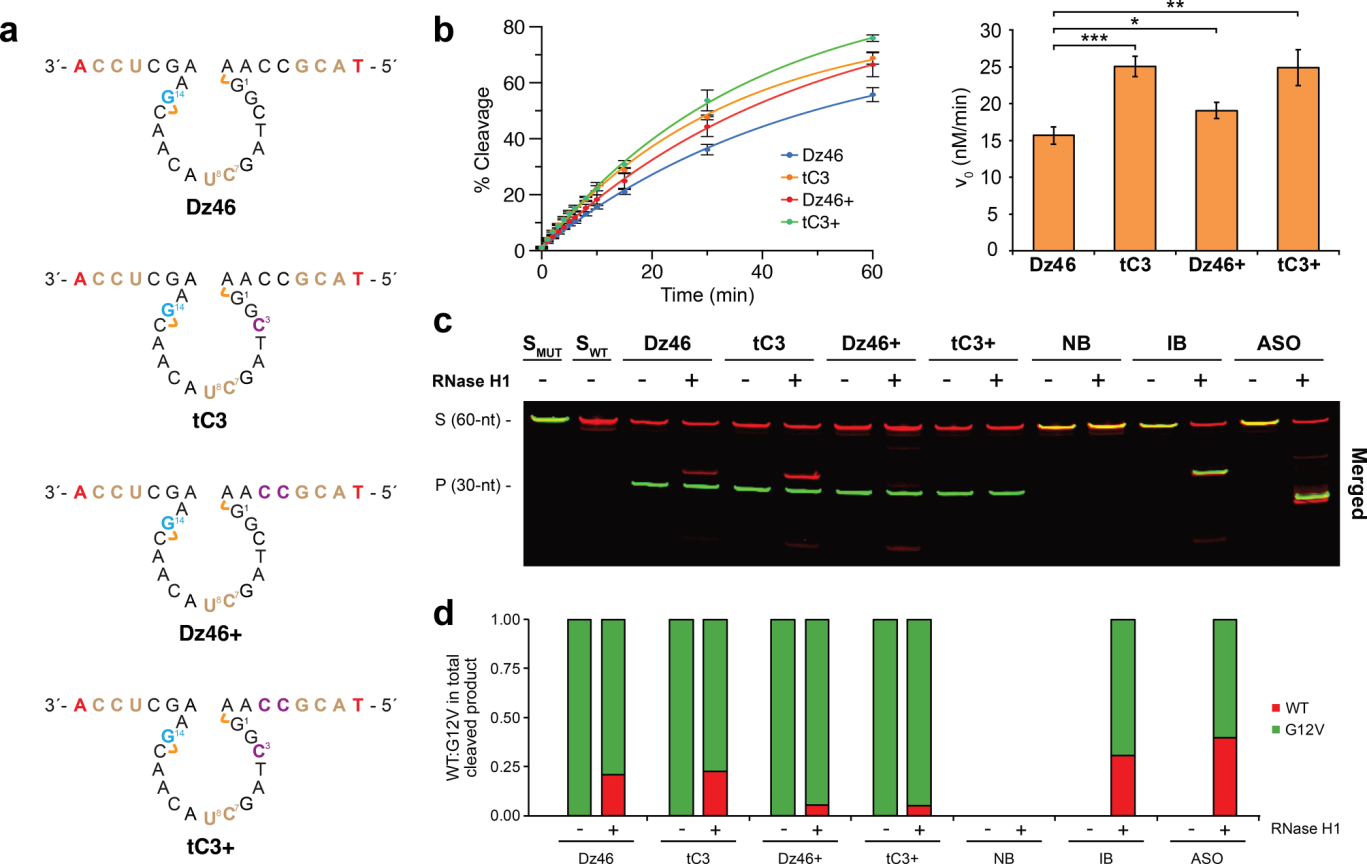
Engineering Dz46 variants with enhanced activity and RNase H1 resistance. (**a**) Engineered Dz46 variants carrying TNA at position dC3 of the catalytic loop (tC3), TNA at −2 and −3 positions of the 5′ binding arm (Dz46+), and TNA in both the catalytic loop and 5′ binding arm (tC3+). (**b**) Kinetic curves and initial velocity bar graphs showing RNA cleavage profiles of Dz46, tC3, Dz46+, and tC3+. Activity was measured under 100:1 (S:E) multiple turnover conditions using a 60-nt RNA substrate with a time course of 0, 1, 2, 3, 4, 5, 6, 8, 10, 15, 30, and 60 min. Data presented as the mean ± standard deviation (*n* = 3). Two-tailed *P*-value determined by Welch’s t-test (*, *P *< .05; **, *P *< .01; ***, *P *< .001). (**c**) Representative denaturing PAGE gel showing RNA cleavage profiles in reactions containing both the wild-type (red) and G12V (green) substrates (1:1) in the absence (−) or presence (+) of 5 ng/μl human RNase H1 after a 30 min incubation (*n* = 2). Merged images of Cy5 (red) and AlexaFluor750 (green) channels are shown. (**d**) Bar graph corresponding to (c) showing the fraction of G12V and WT cleavage in the absence and presence of RNase H1. All reactions were performed under simulated physiological conditions in a buffer containing 1 mM MgCl_2_, 50 mM Tris (pH 7.5), 10 mM NaCl, and 140 mM KCl at 37°C. S: 5′-Cy5-labeled full-length substrate, P: 5′-Cy5-labeled cleavage product. DNAzyme chemistry is color-matched to Fig. 1.

We next asked whether modifications identified in the catalytic loop and binding arms could be combined to establish a TNA-modified Dz46 variant that functioned with enhanced catalytic activity and RNase H1 evasion. We prepared a triply modified Dz46 variant, termed tC3+, containing TNA at position 3 of the catalytic loop as well as positions −2 and −3 of the 5′ binding arm, and compared the activity of this DNAzyme to its predecessors, Dz46, tC3, and Dz46+ (Fig. 3a). The tC3, Dz46+, and tC3+ variants all show improved initial rates over the parent Dz46, with the largest improvement in activity observed for DNAzymes carrying the TNA substitution at position 3 of the catalytic core. The tC3+ variant retained strong catalytic activity (v_0_ = 24.9 ± 2.4 nM/min) comparable to that of tC3 (v_0_ = 25.1 ± 1.4 nM/min), both of which were faster than Dz46 (v_0_ = 15.7 ± 1.2 nM/min) and Dz46+ (v_0_ = 19.1 ± 1.1 nM/min) (Fig. 3b and Supplementary Fig. S6). Interestingly, even though tC3+ and tC3 have similar burst profiles, tC3+ achieved more cleavage over time than tC3 (Fig. 3b and Supplementary Fig. S6). It is possible that the lower binding affinity (∆Tm = –2°C) observed in Dz46 variants with TNA substitution in the binding arms resulted in better product release (Supplementary Fig. S7), improving overall activity under multiple turnover conditions.

In allele-specific assays, tC3+ selectively cleaved the G12V substrate and showed no signs of RNase H1 recognition (Fig. 3c and d and Supplementary Fig. S8), unlike Dz46 and tC3, which produce RNase H1-mediated cleavage products of the wild-type strand. These results establish tC3+ as a lead construct with both high catalytic activity and strong resistance to RNase H1.

### Activity in cultured mammalian cells

**Figure 4.**
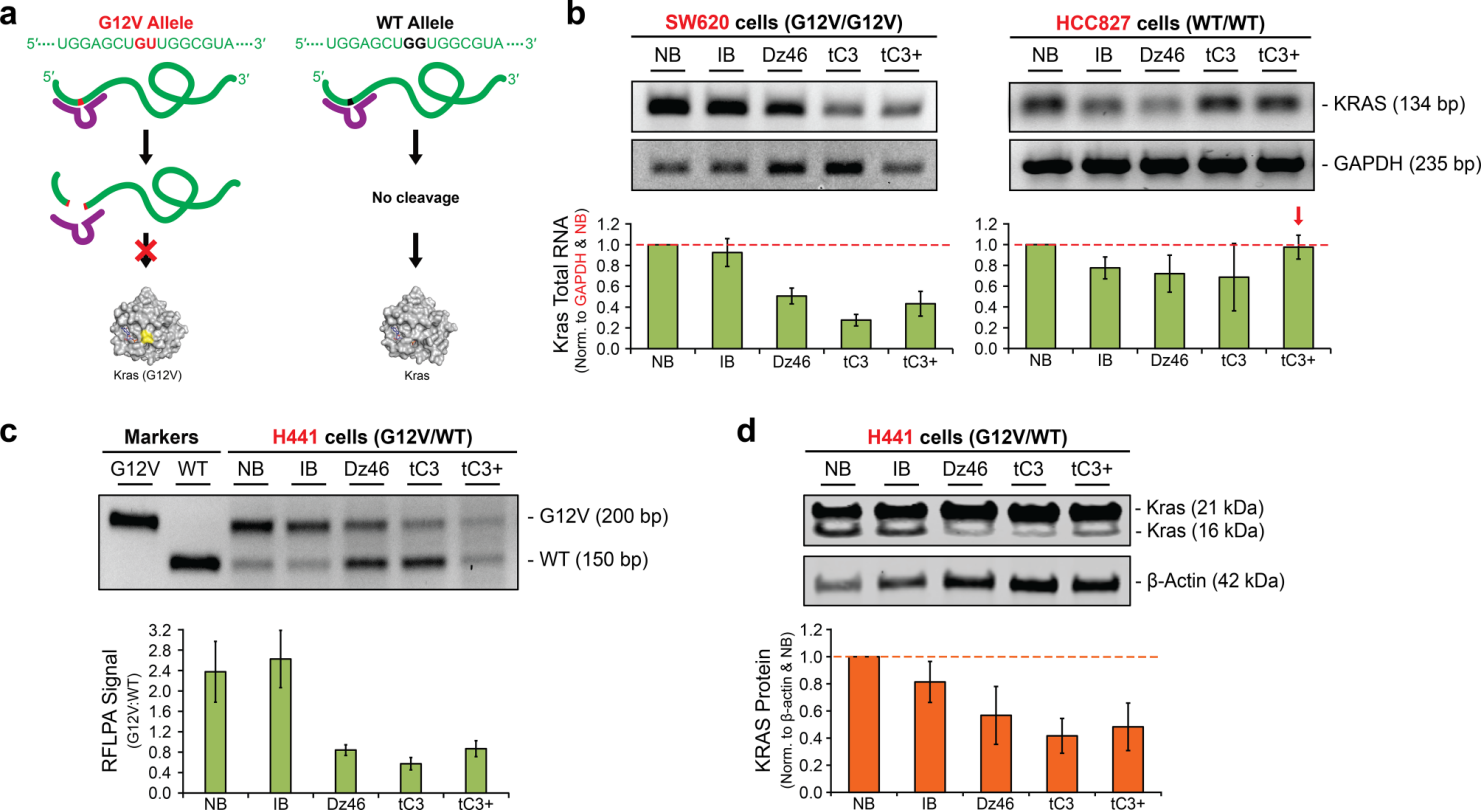
Allele-specific knockdown of KRAS G12V in cells. (**a**) Cartoon representation of allele-specific RNA cleavage. (**b**) Representative agarose gel and corresponding bar graph showing total KRAS levels after DNAzyme treatment of homozygous cell lines for KRAS G12V (SW620, *n* = 3) and KRAS wild-type (HCC827, *n* = 2). Red arrow signifies RNase H1 evasion. (**c**) Representative agarose gel and corresponding bar graph showing allele-specific RNA cleavage of KRAS G12V in H441 cells treated with Dz46 variants. Samples were evaluated by RFLP analysis (*n* = 3). (**d**) Representative immunoblots and corresponding bar graph of total KRAS protein isolated from DNAzyme-treated H441 cells probed with anti-KRAS and β-Actin antibodies (*n* = 4). Data presented as the mean ± standard deviation. All experiments were evaluated 48 h post-transfection and normalized to the NB control.

Recognizing the ability for tC3+ to resist RNase H1 recognition under cell-free conditions, we wished to confirm this activity in a cellular context. Homozygous SW620 (*G12V/G12V*) and HCC827 (*WT/WT*) cell lines exclusively expressing either the G12V or wild-type KRAS genes, respectively, offer a convenient way to test for RNase H1 activity. Since the DNAzymes used in this assay are engineered to recognize the G12V allele, any mRNA knockdown in the HCC827 cell line would be consistent with RNase H1 recognition, while mRNA knockdown in the SW620 cell line could be attributed to either DNAzyme cleavage or RNase H1 (Fig. 4a). At 48 h post-transfection, cells treated with Dz46, tC3, and tC3+ exhibit 27%–51% knockdown of the KRAS G12V allele in SW620 cells (Fig. 4b and Supplementary Fig. S9). However, only tC3+ variant was able to evade RNase H1 recognition in the HCC827 cell line, corroborating our cell-free analysis (Fig. 3).

As expected, NB-treated cells show no activity in either cell line, as this control has no sequence complementarity to the KRAS target. However, close analysis of the data does show an unusual pattern where the IB control appears to selectively knock down KRAS transcript levels in the HCC827 cell line while showing no knockdown activity in the SW620 cell line (Fig. 4b). We attribute this difference to different levels of RNase H1 in the two cell lines, which is consistent with prior work measuring RNase H1 levels across cell lines [25]. Alternatively, it is also possible that the transfection efficiency of the HCC827 cell line is reproducibly higher than the SW620 cell line.

Next, we performed an RFLP analysis of DNAzyme activity in heterozygous KRAS (*G12V*/*WT*) H441 cells expressing both the WT and G12V alleles at a ratio of roughly 1:4 copies per cell, respectively [36]. This assay uses reverse transcription polymerase chain reaction (RT-PCR) to introduce a *Bst*NI restriction site into the wild-type allele, which is then selectively cleaved upon incubation with *Bst*NI restriction endonuclease to yield two bands that are distinguishable by agarose gel analysis. Allele-specific knockdown is then assessed by quantifying the ratio of the G12V to WT band intensities within a treated sample. NB and IB controls show expected G12V:WT ratios that were observed in untreated samples of RFLP analysis (Fig. 4c), where G12V levels are two to three-fold higher than WT levels [25, 35]. All of the active DNzymes tested reduce G12V mRNA levels relative to the wild-type mRNA levels by ~3-fold (Fig. 4c), confirming selective knockdown of the mutant allele. The apparent increase in WT levels observed in the agarose gel (Fig. 4c) is due to changes in the ratio of WT-to-mutant levels in the DNAzyme-treated samples due to knockdown of the G12V allele.

Western blot analysis showed protein-level KRAS knockdown of 43% (Dz46), 58% (tC3), and 52% (tC3+) relative to a non-binding control. Since we were unable to find a satisfactory G12V-specific antibody, this assay was performed using an antibody that recognizes both isoforms of KRAS (16 and 21 kDa; Fig. 4d). Interestingly, cells treated with the IB control show ~20% reduction in total KRAS protein with no concomitant change in KRAS mRNA level (Fig. 4d). In the absence of any observable mRNA knockdown, we attribute this result to a steric block mechanism during protein translation, where binding of the IB control to the mRNA transcript partially inhibits protein translation. Since very few DNAzyme studies evaluate protein knockdown levels in cells, insufficient data is available to comment on how frequently IB controls act as a steric block during protein translation.

### General knockdown of other therapeutic targets

To assess the broader potential of the tC3+ DNAzyme, we extended our analysis to two clinically relevant gene targets: proprotein convertase subtilisin/kexin type 9 (PCSK9) and GATA binding protein 3 (GATA3). These targets were selected for their clinical relevance to hypercholesterolemia and inflammatory diseases, respectively, thereby providing a test of DNAzyme versatility and effectiveness across diverse cellular contexts using a general knockdown (non-allelic) approach [45, 46].

**Figure 5.**
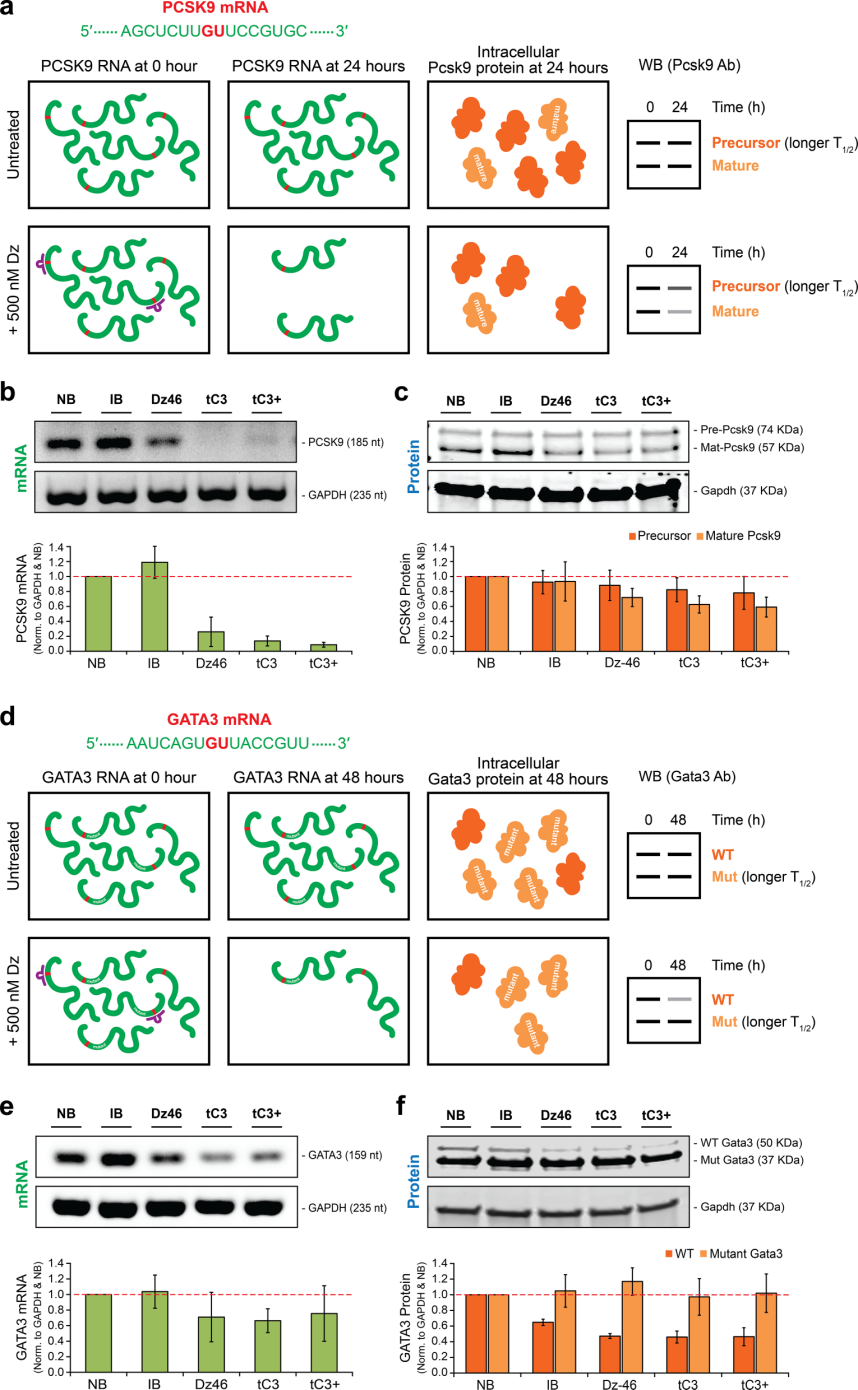
DNAzyme-mediated knockdown of PCSK9 and GATA3. (**a**) Cartoon representation of PCSK9 mRNA targeting and Pcsk9 protein knockdown. (**b**) Representative agarose gel and corresponding bar graph of PCSK9 mRNA knockdown observed in HeLa cells. (**c**) Representative immunoblots and corresponding bar graph of PCSK9 protein knockdown observed in HeLa cells. (**d**) Cartoon representation of GATA3 mRNA targeting and Gata3 protein knockdown. (**e**) Representative agarose gel and corresponding bar graph of GATA3 mRNA knockdown observed in MCF7 cells. (**f**) Representative immunoblots and corresponding bar graph of GATA3 protein knockdown observed in MCF7 cells. Error bars denote ± standard deviation of the mean for three independent experiments. All data were normalized to the NB control. Abbreviations: NB, non-binder; IB, inactive binder; Dz46, DNAzyme 46; tC3, Dz46 with tC3 modification; tC3+, Dz46 with tC3 and TNA in the 5′ binding arm.

PCSK9 is a liver-secreted protease that plays a central role in cholesterol homeostasis by promoting the degradation of low-density lipoprotein receptors (LDLR) on hepatocyte surfaces [47]. Inhibition of PCSK9 increases LDLR availability, leading to enhanced clearance of circulating LDL cholesterol, a mechanism that underpins the success of PCSK9-targeted monoclonal antibody therapies [48]. However, with the FDA approval of inclisiran [49], a first-in-class siRNA drug to lower cholesterol by targeting PCSK9, it has been demonstrated that oligonucleotide-based approaches may offer a more cost-effective and tissue-targetable alternative [16]. We designed constructs targeting a GU dinucleotide junction in exon 13 of PCSK9 mRNA (nucleotides 2710–2711), a canonical cleavage site for the 10-23 catalytic motif. Using HeLa cells as a model system, we measured mRNA levels by RT-PCR 24 h post-transfection (Fig. 5a and b and Supplementary Fig. S9). All three active DNAzymes, Dz46, tC3, and tC3+, exhibited robust mRNA knockdown, reducing PCSK9 transcript levels by 74%, 86%, and 99%, respectively, relative to the non-binding control. Immunoblot analysis confirmed the functional outcome of this knockdown, with mature PCSK9 protein levels decreasing by 30%–40%, with the greatest effect observed for tC3+ (Fig. 5c). This result demonstrates that tC3+ is capable of high-efficiency gene silencing at both the RNA and protein levels for targets beyond KRAS.

GATA3, a zinc finger transcription factor, is a master regulator of Th2 cell differentiation and immune activation, and is also implicated in the pathogenesis of several diseases, including asthma [50], breast cancer [51], and certain leukemias [52]. Elevated GATA3 expression has been observed in the airway epithelium of patients with severe asthma, where it contributes to chronic inflammation and cytokine dysregulation [53]. GATA3 is also highly expressed in luminal breast cancer, where it exists as both a full-length 50 kDa protein and a truncated 37 kDa isoform resulting from a frameshift mutation [54]; the latter displays increased stability due to its prolonged half-life (>8 h) [55]. To explore whether our DNAzymes could knock down GATA3, we designed constructs targeting a GU junction in exon 6 of the GATA3 transcript (nucleotides 2490–2491) and transfected MCF7 breast cancer cells with Dz46, tC3, and tC3+ variants (Fig. 5d and Supplementary Fig. S9).

At the mRNA level, GATA3 knockdown was modest but consistent, with reductions of 29% (Dz46), 36% (tC3), and 24% (tC3+) compared to the non-binding control (Fig. 5e). Notably, these reductions were more pronounced at the protein level, wherein the full-length GATA3 protein was diminished by ∼55% across all active variants (Fig. 5f). The truncated isoform was not reduced, potentially due to its long half-life [55]. Interestingly, even the IB control showed a 35% reduction in GATA3 protein levels, suggesting that IB control could once again be acting as a steric block during protein translation.

## Discussion

We demonstrate that the incorporation of TNA into Dz46 facilitates enhanced catalytic activity and strong RNase H1 evasion. This advance addresses two important limitations of RNA-cleaving DNAzymes: (i) modest activity under physiological conditions and (ii) unintended activation of cellular RNase H1, thereby establishing a new framework for engineering autonomous gene silencing agents based on XNA chemistry.

The systematic TNA walk across the catalytic core revealed unexpected hotspots of tolerance, particularly at positions C3 and A9, which were not known previously as permissive sites for chemical modification in the 10-23 DNAzyme framework. The improved activity observed with TNA substitution at dC3 suggests a beneficial alteration in the local structural environment of the DNAzyme, possibly enhancing metal ion coordination or stabilizing the catalytic geometry. Importantly, the increased activity of the tC3 variant was preserved in longer RNA substrates and in cellular knockdown assays, validating its functional relevance beyond cell-free models. These findings expand the repertoire of chemical modifications compatible with the 10-23 motif and open new avenues for enhancing DNAzyme performance through nucleic acid engineering [17].

Equally notable is the strategic placement of adjacent TNA residues in the 5′ binding arm, which was sufficient to significantly reduce RNase H1-mediated cleavage without sacrificing catalytic efficiency. This outcome contrasts with earlier attempts to reduce RNase H1 recognition through competitive models that enhance catalytic turnover [25]. Our data confirm that RNase H1 cleavage of the wild-type KRAS allele can be abrogated through precise modification of the binding arm, enabling true allele-specific discrimination against the G12V oncogenic variant. This effect was maintained in cellular assays, where tC3+ achieved selective KRAS knockdown in heterozygous and homozygous mutant cell lines, with essentially no activity against the wild-type allele.

The generalizability of the tC3+ architecture was tested against two additional therapeutic targets, PCSK9 and GATA3, which differ significantly in their expression, biological role, and molecular complexity. The marked knockdown of PCSK9 at both the RNA and protein levels positions tC3+ as a candidate for hepatic gene silencing applications, particularly in the context of cholesterol-lowering therapies. For GATA3, while mRNA knockdown was more moderate, protein-level reductions were substantial, particularly for the full-length isoform. This disconnect between RNA and protein knockdown levels highlights the nuanced relationship between protein target stability, transcript accessibility, and DNAzyme activity in different cellular environments, and underscores the importance of protein-level validation in functional studies.

A consistent theme across all experimental systems is the superior performance of the tC3+ variant within the set of DNAzymes tested. By integrating catalytic enhancement and RNase H1 evasion into a single construct, this variant overcomes limitations typically encountered when modifications are applied in isolation, e.g. using structure-based approaches. These results affirm the utility of combining iterative design methods with nucleic acid chemistry to develop next-generation DNAzymes with improved pharmacodynamic properties. The demonstration of efficacy across three clinically relevant targets using allele-specific and general knockdown activity further supports the therapeutic potential of this strategy.

In summary, our findings establish TNA as a powerful tool for engineering DNAzymes with enhanced intracellular activity and allele-specific precision. The modular design of tC3+ supports its adaptation to a broad range of gene targets, paving the way for future studies focused on pharmacokinetics, biodistribution, and *in vivo* efficacy. More broadly, our work highlights the value of XNA chemistry in advancing oligonucleotide therapeutics and suggests that further exploration of non-canonical sugar backbones may yield additional gains in therapeutic utility.

## Supplementary Material

gkaf1476_Supplemental_File

## Data Availability

All data are available upon reasonable request.
