# Supplementary material for: Allele-specific knockdown by an engineered DNAzyme capable of RNase H1 evasion
Source: Nucleic Acids Res. 2026 Jan 6;54(1):gkaf1476. doi: 10.1093/nar/gkaf1476 (PMC12774632; doi:10.1093/nar/gkaf1476)
Supplement: gkaf1476_Supplemental_File [file gkaf1476_supplemental_file.pdf]

Supplementary Information

Allele-Specific Knockdown by an Engineered DNAzyme Capable of RNase H1 Evasion

Erica M. Lee[1†], Kim Nguyen[1†], Noah A. Setterholm[1], Turnee N. Malik[1], and John C. Chaput[1-4*]

[†]These authors contributed equally

[1]Department of Pharmaceutical Sciences, University of California, Irvine, CA 92697, USA
[2]Department of Chemistry, University of California, Irvine, CA 92697, USA
[3]Department of Molecular Biology and Biochemistry, University of California, Irvine, CA 92697, USA
[4]Department of Chemical and Biomolecular Engineering, University of California, Irvine, CA 92697, USA

Table of Contents

Supplementary Table 1. Dz46 variants used in the TNA walk.

Supplementary Table 2. Dz46 variants with chemical modification at terminal and internal positions.

Supplementary Table 3. DNAzymes used in KRAS G12V knockdown experiments.

Supplementary Table 4. DNAzymes used in PCSK9 knockdown experiments.

Supplementary Table 5. DNAzymes used in GATA3 knockdown experiments.

Supplementary Table 6. Control oligonucleotides.

Supplementary Table 7. RNA substrates.

Supplementary Table 8. Primer oligos used in RT-PCR.

Supplementary Table 9. Primer oligos used in PCR-RFPLA.

Supplementary Figure 1. Validation of product produced by Dz cleavage.

Supplementary Figure 2. TNA walk under different conditions.

Supplementary Figure 3. RNA cleavage activity of Dz46, tC3, and tA9 on different length substrates.

Supplementary Figure 4. Signal bleed through of AlexaFluor750 from the 800 to the 700 channel on the Odyssey CLx Imaging System (LI-COR).

Supplementary Figure 5. Modulating RNase H activity using TNA modifications in the binding arms.

Supplementary Figure 6. RNA cleavage activity of Dz46 and its variants carrying TNA modifications.

Supplementary Figure 7. Melting temperatures of Dz46 and its variants with TNA incorporations.

Supplementary Figure 8. RNase H activity of final chosen Dz46 variants carrying TNA modifications in the catalytic core and binding arms.

Supplementary Figure 9. Human KRAS, PCSK9 and GATA3 targets.

Supplementary Table 1. Dz46 variants used in the TNA walk.

DNAzyme ID	Sequence (5' → 3'; IDT nomenclature)	Calculated Mass (Da)	Observed Mass (Da)	Δ Mass (Da)
tA0	+TmAmCmGCCA**tA***GGCTAGmCmUACAAC*/i2MOErG/AAGCmUmCmC+A	9514.17	9518.65	4.48
tG1	+TmAmCmGCCAA***tG**GCTAGmCmUACAAC*/i2MOErG/AAGCmUmCmC+A	9514.17	9520.00	5.83
tG2	+TmAmCmGCCAA*G**tG**CTAGmCmUACAAC*/i2MOErG/AAGCmUmCmC+A	9514.17	9521.91	7.74
tC3	+TmAmCmGCCAA*GG**tC**TAGmCmUACAAC*/i2MOErG/AAGCmUmCmC+A	9514.17	9520.30	6.13
tT4	+TmAmCmGCCAA*GGC**tT**AGmCmUACAAC*/i2MOErG/AAGCmUmCmC+A	9514.17	9520.69	6.52
tA5	+TmAmCmGCCAA*GGCT**tA**GmCmUACAAC*/i2MOErG/AAGCmUmCmC+A	9514.17	9522.65	8.48
tG6	+TmAmCmGCCAA*GGCTA**tG**mCmUACAAC*/i2MOErG/AAGCmUmCmC+A	9514.17	9522.44	8.27
tC7	+TmAmCmGCCAA*GGCTAG**tC**mUACAAC*/i2MOErG/AAGCmUmCmC+A	9484.14	9492.35	8.21
tT8	+TmAmCmGCCAA*GGCTAGmC**tT**ACAAC*/i2MOErG/AAGCmUmCmC+A	9498.16	9506.26	8.10
tA9	+TmAmCmGCCAA*GGCTAGmCmU**tA**CAAC*/i2MOErG/AAGCmUmCmC+A	9514.17	9519.74	5.57
tC10	+TmAmCmGCCAA*GGCTAGmCmUA**tC**AAC*/i2MOErG/AAGCmUmCmC+A	9514.17	9521.08	6.91
tA11	+TmAmCmGCCAA*GGCTAGmCmUAC**tA**AC*/i2MOErG/AAGCmUmCmC+A	9514.17	9521.48	7.31
tA12	+TmAmCmGCCAA*GGCTAGmCmUACA**tA**C*/i2MOErG/AAGCmUmCmC+A	9514.17	9521.23	7.06
tC13	+TmAmCmGCCAA*GGCTAGmCmUACAA**tC***/i2MOErG/AAGCmUmCmC+A	9514.17	9521.05	6.88
tG14	+TmAmCmGCCAA*GGCTAGmCmUACAAC***tG** AAGCmUmCmC+A	9440.09	9446.36	6.27
tA15	+TmAmCmGCCAA*GGCTAGmCmUACAAC*/i2MOErG/**tA**AGCmUmCmC+A	9514.17	9520.45	6.28
tA16	+TmAmCmGCCAA*GGCTAGmCmUACAAC*/i2MOErG/A**tA**GCmUmCmC+A	9514.17	9521.41	7.24

TNA (t); LNA (+); 2'-O-methoxyribonucleic acid (m); phosphorothioate linkage (*); 2'-O- methoxyethylribonucleic acid (/i2MOEr/).

Supplementary Table 2. Dz46 variants with chemical modification at the terminal and internal positions.

DNAzyme ID	Sequence (5' → 3' or 3' → 2'; IDT nomenclature)	Calculated Mass (Da)	Observed Mass (Da)	Δ Mass (Da)
Dz46+	+TmAmCmG**tCtC**AA*GGCTAGmCmUACAAC*/i2MOErG/AAGCmUmCmC+A	9500.15	9502.51	2.36
NS580	**tT**mAmCmGCCAA*GGCTAGmCmUACAAC*/i2MOErG/AAGCmUmCmC**tA**	9444.13	9450.83	6.70
NS580+	**tT**mAmCmG**tCtC**AA*GGCTAGmCmUACAAC*/i2MOErG/AAGCmUmCmC**tA**	9416.09	9424.63	8.54

TNA (t); LNA (+); 2'-O-methoxyribonucleic acid (m); phosphorothioate linkage (*); 2'-O- methoxyethylribonucleic acid (/i2MOEr/).

Supplementary Table 3. DNAzymes used in KRAS G12V knockdown experiments.

DNAzyme ID	Sequence (5' → 3'; IDT nomenclature)	Calculated Mass (Da)	Observed Mass (Da)	∆ Mass (Da)
Dz46	+TmAmCmGCCAA*GGCTAGmCmUACAAC*/i2MOErG/AAGCmUmCmC+A	9528.23	9533.33	5.1
tC3 (Dz46 ver.)	+TmAmCmGCCAA*GGtCTAGmCmUACAAC*/i2MOErG/AAGCmUmCmC+A	9514.17	9520.30	6.13
tC3+ (Dz46 ver.)	+TmAmCmGtCtCAA*GGtCTAGmCmUACAAC*/i2MOErG/AAGCmUmCmC+A	9486.13	9493.73	7.60
Inactive Dz46	+TmAmCmGCCAA*GGCCATmCmUACAAC*/i2MOErG/A AGCmUmCmC+A	9488.20	9494.35	6.15

TNA (t); LNA (+); 2'-O-methoxyribonucleic acid (m); phosphorothioate linkage (*); 2'-O- methoxyethylribonucleic acid (/i2MOEr/).

Supplementary Table 4. DNAzymes used in PCSK9 knockdown experiments.

DNAzyme ID	Sequence (5' → 3'; IDT nomenclature)	Calculated Mass (Da)	Observed Mass (Da)	∆ Mass (Da)
TNM243	+GmCmAmCGGAA*GGCTAGmCmUACAAC*/i2 MOErG/AAAGmAmGmC+T	9671.36	9675.10	3.74
tC3 (TNM243 ver.)	+GmCmAmCGGAA*GGtCTAGmCmUACAAC*/i2 MOErG/AAAGmAmGmC+T	9657.34	9660.76	3.42
tC3+ (TNM243 ver.)	+GmCmAmCtGtGAA*GGtCTAGmCmUACAAC*/i2 MOErG/AAAGmAmGmC+T	9629.30	9632.71	3.41
Inactive TNM243	+GmCmAmCGGAA*GGCCATmCmUACAAC*/i2 MOErG/AAAGmAmGmC+T	9631.33	9634.29	2.96

TNA (t); LNA (+); 2'-O-methoxyribonucleic acid (m); phosphorothioate linkage (*); 2'-O- methoxyethylribonucleic acid (/i2MOEr/).

Supplementary Table 5. DNAzymes used in GATA3 knockdown experiments.

DNAzyme ID	Sequence (5' → 3'; IDT nomenclature)	Calculated Mass (Da)	Observed Mass (Da)	∆ Mass (Da)
KN155	+AmAmCmGGTAA*GGCTAGmCmUACAAC*/i2 MOErG/AACTmGmAmU+T	9622.33	9625.98	3.65
tC3 (KN155 ver.)	+AmAmCmGGTAA*GGtCTAGmCmUACAAC*/i2 MOErG/AACTmGmAmU+T	9608.31	9613.88	5.57
tC3+ (KN155 ver.)	+AmAmCmGtGtTAA*GGtCTAGmCmUACAAC*/i2 MOErG/AACTmGmAmU+T	9580.26	9583.11	2.85
Inactive KN155	+AmAmCmGGTAA*GGCCATmCmUACAAC*/i2 MOErG/AACTmGmAmU+T	9582.30	9587.37	5.07

TNA (t); LNA (+); 2'-O-methoxyribonucleic acid (m); phosphorothioate linkage (*); 2'-O- methoxyethylribonucleic acid (/i2MOEr/).

Supplementary Table 6. Control oligonucleotides.

DNAzyme ID	Sequence (5' → 3'; IDT nomenclature)	Calculated Mass (Da)	Observed Mass (Da)	∆ Mass (Da)
Non-binder (used in RNase H1 assays)	+AmGmGmUGACA*GGCTAGmCmUACAAC*/i2MOErG/A TATmAmGmA+A	9671.38	9675.84	4.46
Non-binder (used in cellular assays)	+AmCmCmGCGCA*GGCTAGmCmUACAAC*/i2MOErG/A CCGmUmUmU+G	9523.17	9528.21	5.04
ASO	GCCTACGCCAACAGCTCCAACT	-	-	-

TNA (t); LNA (+); 2'-O-methoxyribonucleic acid (m); phosphorothioate linkage (*); 2'-O- methoxyethylribonucleic acid (/i2MOEr/).
ASO was ordered from IDT.

Supplementary Table 7. RNA substrates.

RNA substrates	Sequence (5' → 3'; IDT nomenclature)
16 nt –G12V KRAS (NCBI: NM_ 004985, human GTPase KRAS mRNA variant b, (positions 217-232: G225U mutation, codon 12 GGU to GUU)	/5Cy5/rUrGrGrArGrCrUrGrUrUrGrGrCrGrUrA
60 nt –G12V KRAS (NCBI: NM_ 004985, human GTPase KRAS mRNA variant b, (positions 195-254: G225U mutation, codon 12 GGU to GUU)	/5Cy5/rCrUrGrArArUrArUrArArArCrUrUrGrU rGrGrUrArGrUrUrGrGrArGrCrUrGrUrUrGrGr CrGrUrArGrGrCrArArGrArGrUrGrCrCrUrUrG rArCrG rArUrArC
60 nt- G12V KRAS (5'Alexa Fluor 750)	/5Alex750N/rCrUrGrArArUrArUrArArArCrUrUr GrUrGrGrUrArGrUrUrGrGrArGrCrUrGrUrUrG rGrCrGrUrArGrGrCrArArGrArGrUrGrCrCrUrU rGrArCrGrArUrArC
60 nt –WT KRAS (NCBI: NM_ 004985, human GTPase KRAS mRNA variant b, (positions 195-254)	/5Cy5/rCrUrGrArArUrArUrArArArCrUrUrGrU rGrGrUrArGrUrUrGrGrArGrCrUrGrGrUrGrGr CrGrUrArGrGrCrArArGrArGrUrGrCrCrUrUrG rArCrG rArUrArC

Oligonucleotides were ordered from IDT.

Supplementary Table 8. Primer oligos used in RT-PCR.

Oligo #	Sequence (5' → 3')	Amplicon (size)
KN469F	GGCCTGCTGAAAATGACTGAATATAAAC	KRAS (134 nt)
KN470R	ACAAGATTTACCTCTATTGTTGGATCATATTCG	KRAS
KN60F	ACCATCTTCCAGGAGCGAGATCCCTC	GAPDH (235 nt)
KN61R	TGCAGGAGGCATTGCTGATGATCTTGA	GAPDH
KN601F	TTTCACCATTCAAACAGGTCGAGCTG	PCSK9 (185 nt)
KN602R	TCCCTGCAGCCCCTACC	PCSK9
KN603F	GTTGTAGGCGAATCATTTGTTCAAAGCTG	GATA3 (159 nt)
KN604R	CAAACAACAATTACAGGGACTTGTTCACAAAG	GATA3

Oligonucleotides were ordered from IDT.

Supplementary Table 9. Primer oligos used in PCR-RFPLA.

Oligo #	Sequence (5' → 3')	Amplicon (size)
KN484F	TTATTATAAATAATGACTGAATATAAACTTGTGGTAGTTGGAcCT	KRAS (200 nt)
KN483R	TCCTCTTGACCTGCTGTGTCG	KRAS

Lower case "c" in KN484F denotes a mutation introduced in the primer to create *BstN*I recognition sequence. Oligonucleotides were ordered from IDT.

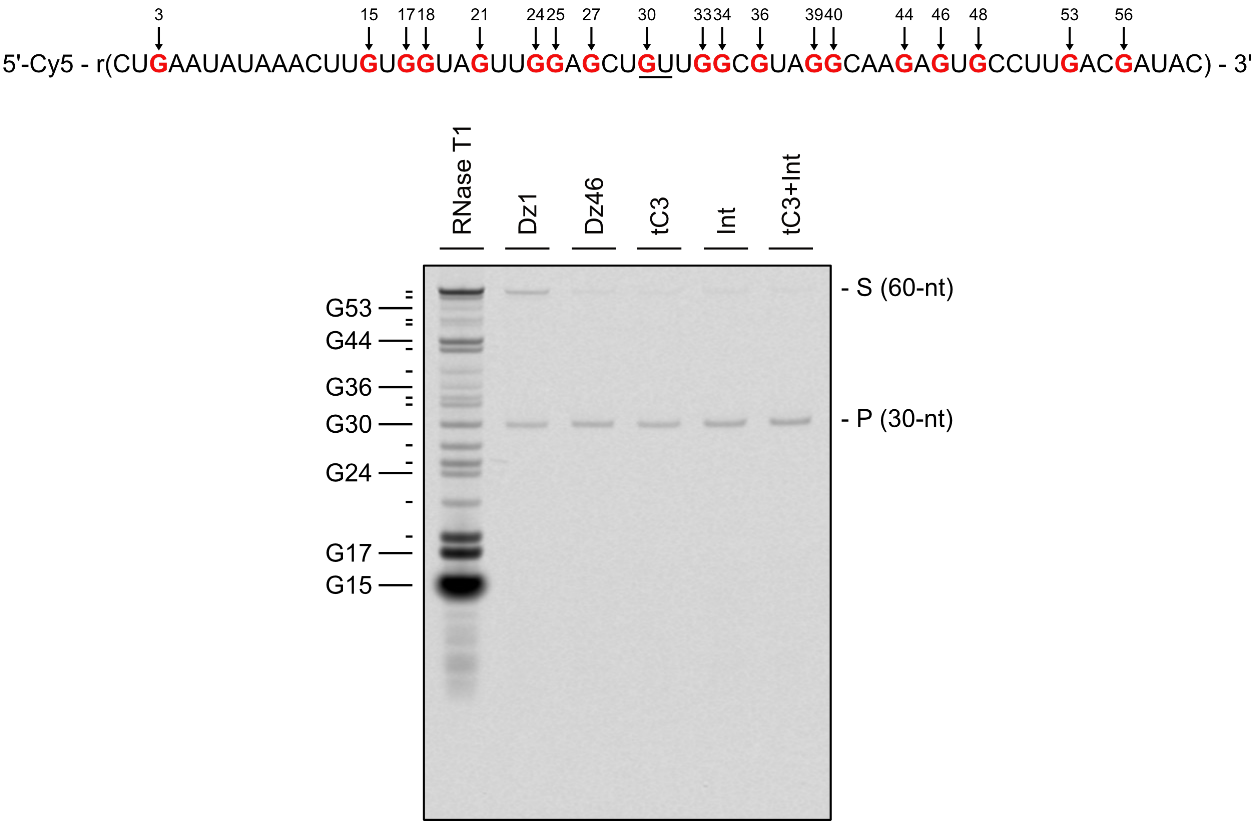

Supplementary Figure 1. Product Validation by Dz cleavage. Depiction of full 60-nt G12V RNA substrate showing the 19 RNase T1 cut sites after G residues (red) and desired G-U cleavage junction (underlined). Denaturing PAGE gel showing product bands produced by Dz1 (unmodified 10-23), Dz46, tC3, Int, and tC3+Int alongside an RNA ladder produced by RNase T1. DNAzyme-catalyzed reactions were performed as 1:1 (S:E) single turnover in 1 mM $MgCl_2$, 50 mM Tris (pH 7.5), 10 mM NaCl, and 140 mM KCl at 37°C with 30-min incubation. The RNase T1 digestion was performed using 1 unit of RNase T1 in 50 mM Tris (pH 7.5), 10 mM NaCl, and 140 mM KCl at 24°C following a 1-min incubation. S: 5′-Cy5-labeled full-length substrate, P: 5′-Cy5-labeled cleavage product.

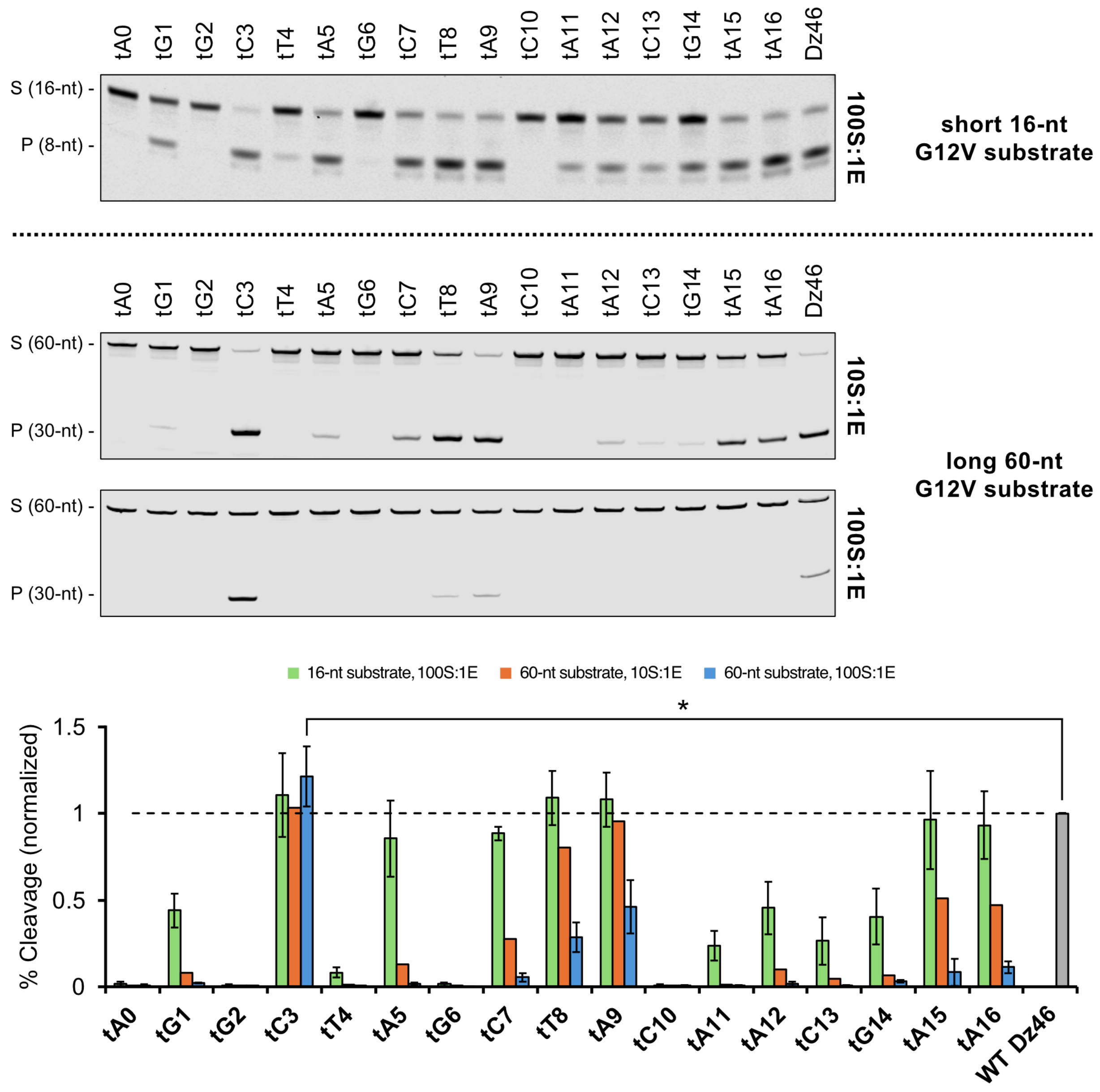

Supplementary Figure 2. TNA walk of the catalytic loop region. Representative denaturing PAGE gel and corresponding bar graph showing RNA cleavage activity of a G12V RNA substrate after 30 min under different multiple turnover conditions on the short 16-nt substrate, 100S:1E (n=3), and long 60-nt substrate at both 10S:1E (n=1) and 100S:1E (n=3 for all constructs except for tC3 and Dz46 which have n=6). RNA cleavage activity (% cleavage) was normalized to Dz46. Data presented as the mean ± standard deviation. Two-tailed p-value determined by Welch's t-test (*, $p<0.05$). All reactions were performed under simulated physiological conditions in buffer containing 1 mM $MgCl_2$, 50 mM Tris (pH 7.5), 10 mM NaCl, and 140 mM KCl at 37°C. S: 5′-Cy5-labeled full-length substrate, P: 5′-Cy5-labeled cleavage product.

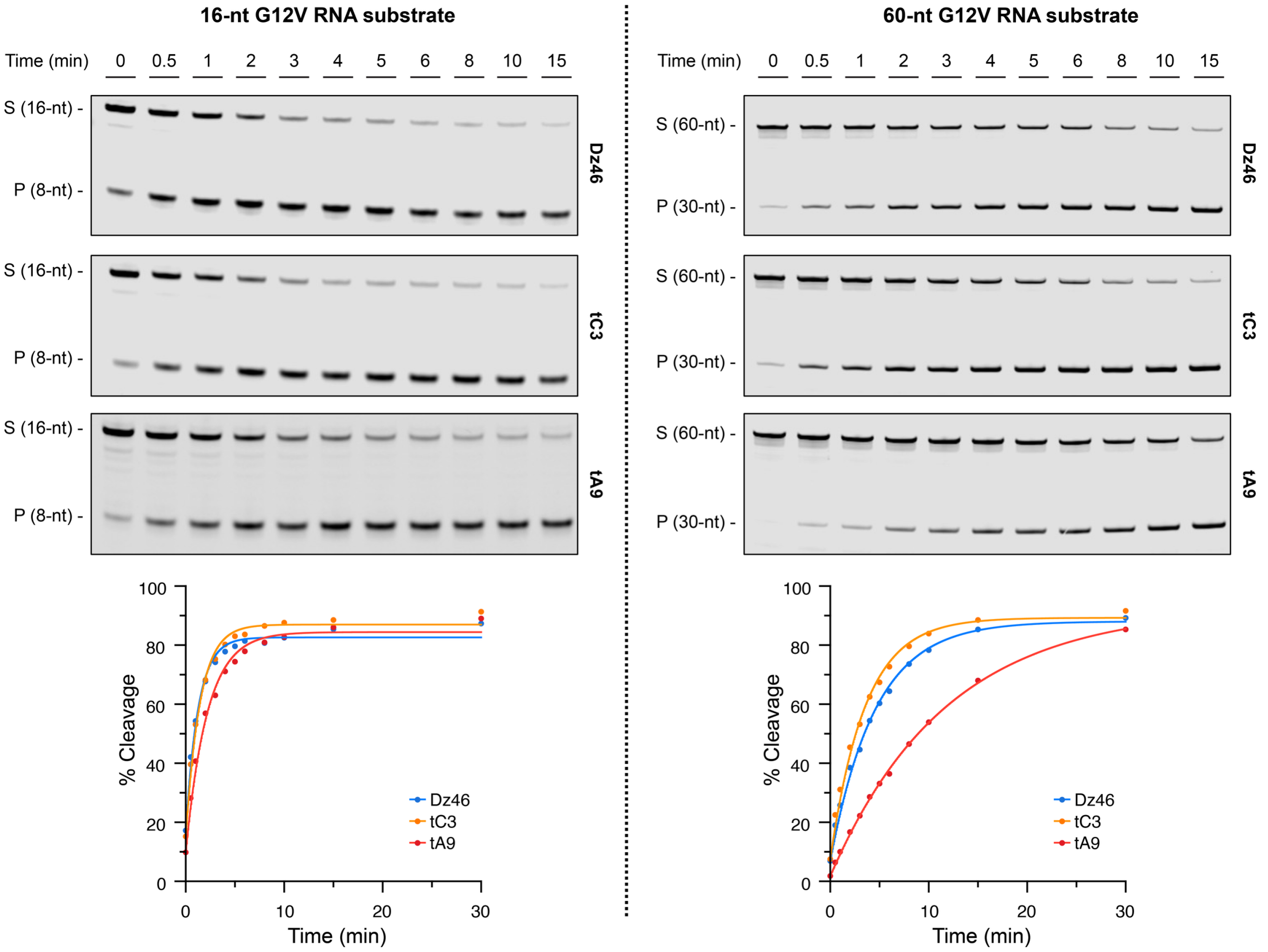

Supplementary Figure 3. RNA cleavage activity of Dz46, tC3, and tA9 on different length substrates. Representative denaturing PAGE gel and corresponding kinetic curves showing 10:1 (S:E) multiple turnover RNA cleavage activity of a short 16-nt RNA substrate versus long 60-nt RNA substrate over the course of 15 min (n=1). All reactions were performed under simulated physiological conditions in buffer containing 1 mM $MgCl_2$, 50 mM Tris (pH 7.5), 10 mM NaCl, and 140 mM KCl at 37°C.

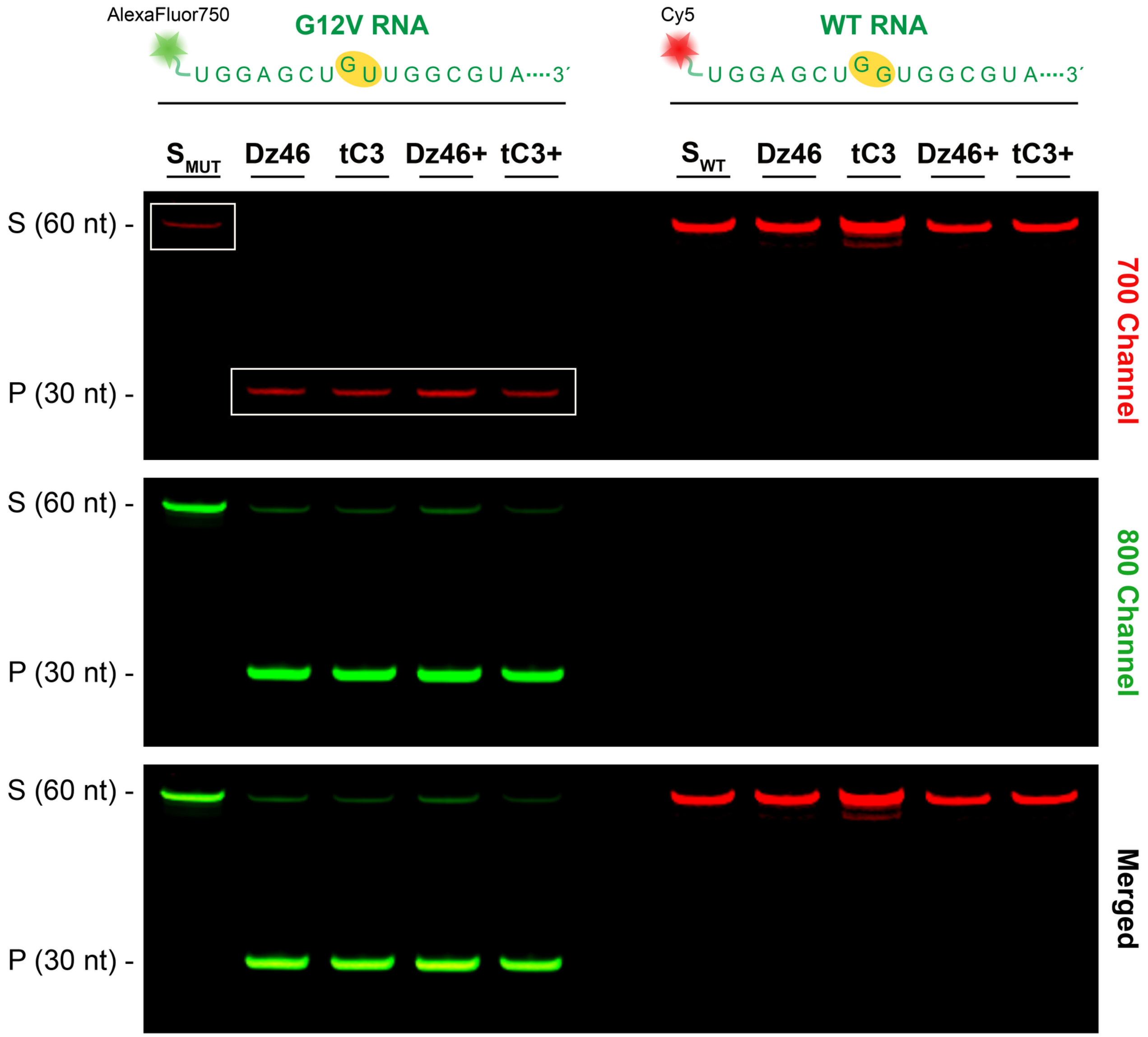

Supplementary Figure 4. Signal bleed through of AlexaFluor750 from the 800 to the 700 channel on the Odyssey CLx Imaging System (LI-COR). Denaturing PAGE gel showing RNA cleavage profiles in 1:2 (S:E) single turnover reactions containing either the 60-nt G12V (green) or wild-type (red) substrates after a 30 min incubation. Individual 700 (red) and 800 (green) channels, as well as the merged image, are shown. Bleed through is observed in the P30 and P60 bands for AlexaFluor750 (green) (white box). Reactions performed under simulated physiological conditions in buffer containing 1 mM $MgCl_2$, 50 mM Tris (pH 7.5), 10 mM NaCl, and 140 mM KCl at 37°C. S: 5'-labeled full-length substrate, P: 5'-labeled cleavage product.

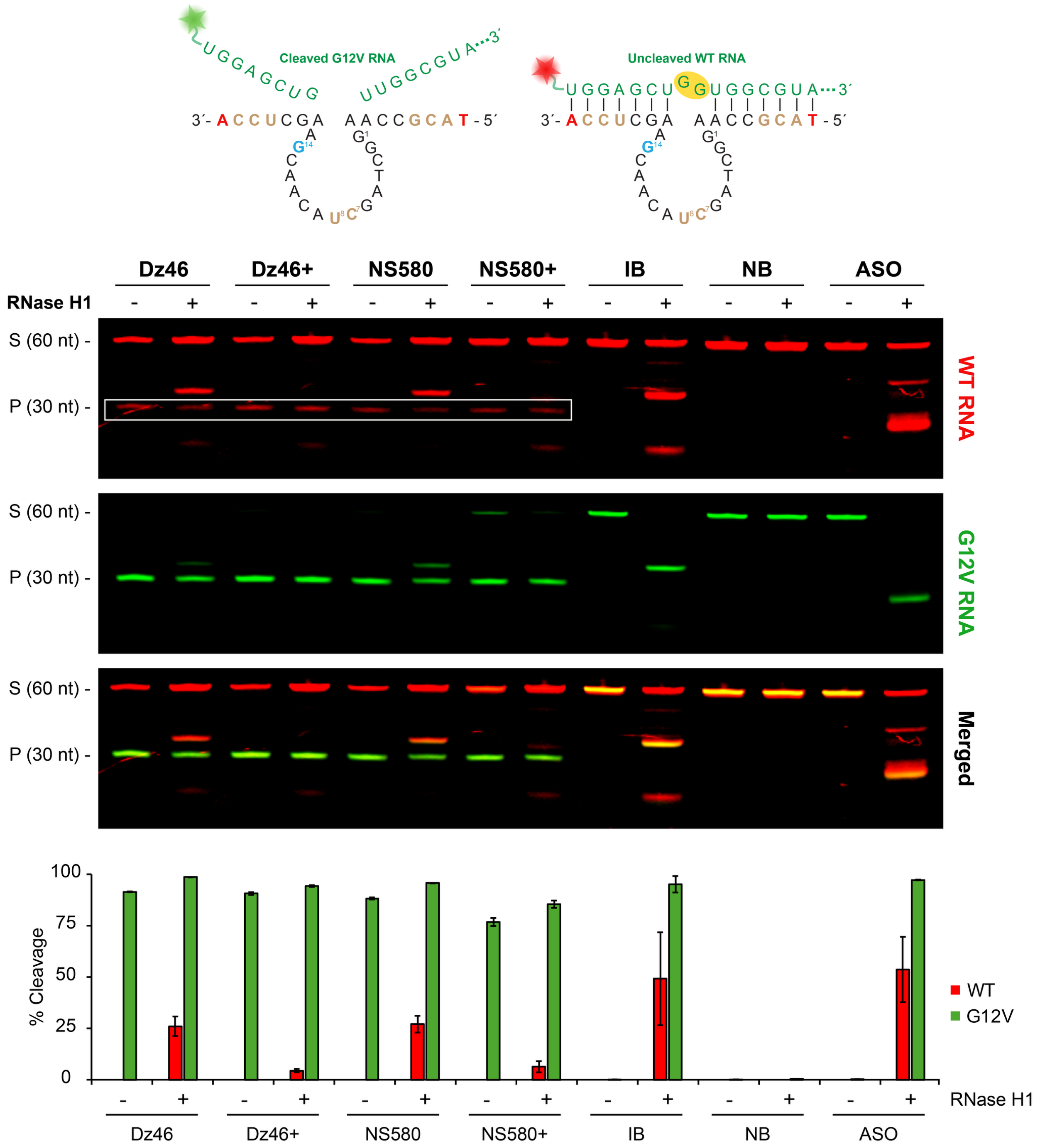

Supplementary Figure 5. Modulating RNase H activity using TNA modifications in the binding arms. Cartoon representation of allele-specific G12V RNA cleavage and representative denaturing PAGE gel showing RNA cleavage profiles in reactions containing both the wild-type (red) and G12V (green) substrates (1:1) in the absence (-) or presence (+) of 5 ng/μL human RNase H1 after a 30 min incubation (n=2). Individual Cy5 (red) and AlexaFluor750 (green) channels, as well as the merged image, are shown. The corresponding bar graph shows the percent cleavage of WT (red) and G12V (green) substrate. The band at P30 was excluded from the analysis due to signal bleed through in the 700 nm channel (white box). Reactions performed under simulated physiological conditions in buffer containing 1 mM $MgCl_2$, 50 mM Tris (pH 7.5), 10 mM NaCl, and 140 mM KCl at 37°C. S: 5'-labeled full-length substrate, P: 5'-labeled cleavage product.

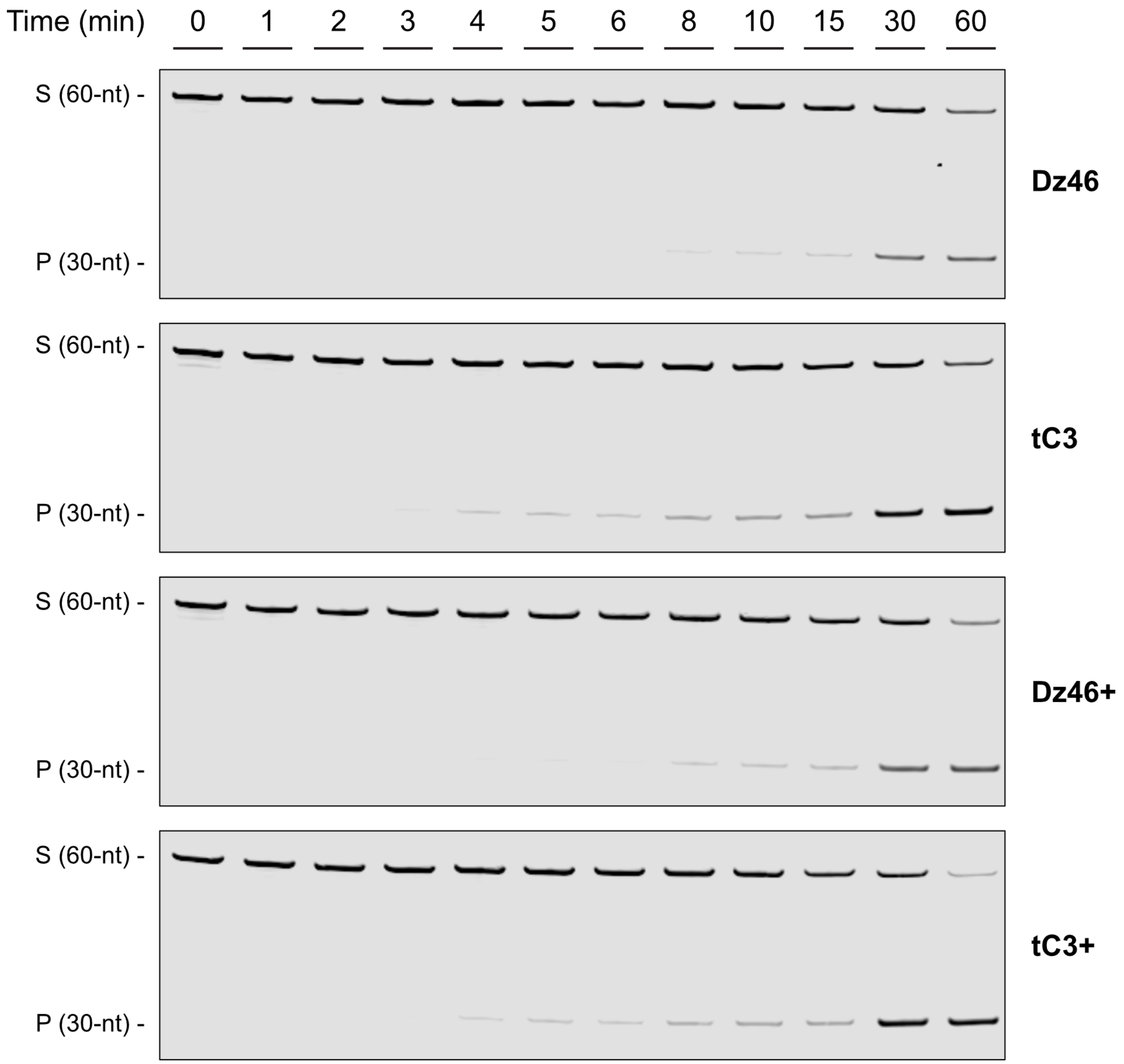

Supplementary Figure 6. Representative gels showing RNA cleavage activity of Dz46 and its variants carrying TNA modifications. Representative denaturing PAGE gel showing 100:1 (S:E) multiple turnover RNA cleavage activity of a long 60-nt RNA substrate over the course of 60 min (n=2). Corresponding kinetic curves and initial velocity bar graphs shown in Figure 3b. All reactions were performed under simulated physiological conditions in buffer containing 1 mM $MgCl_2$, 50 mM Tris (pH 7.5), 10 mM NaCl, and 140 mM KCl at 37°C. S: 5'-labeled full-length substrate, P: 5'-labeled cleavage product.

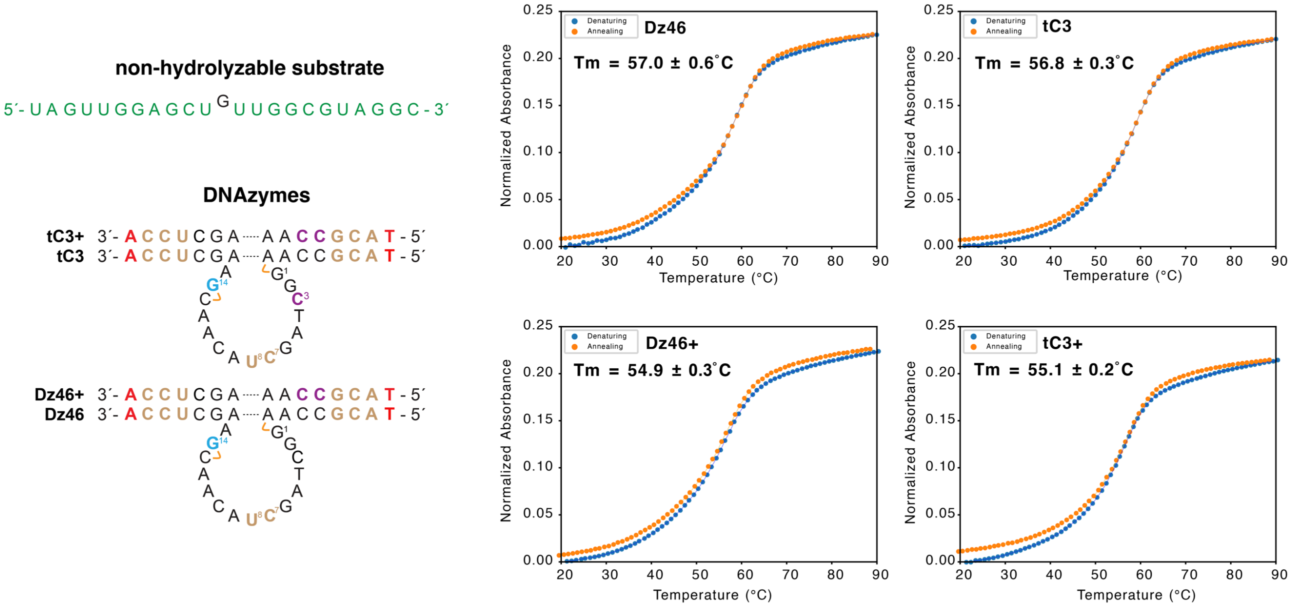

Supplementary Figure 7. Melting temperatures of Dz46 and its variants with TNA incorporations. An uncleavable 21-nt RNA substrate with a 2′-deoxyribose G replacement at the G-U dinucleotide cleavage junction was used for melt measurements. All melts were performed with 1:1 oligonucleotide stoichiometry (1 μM enzyme + 1 μM RNA substrate) in 1 M NaCl, 50 mM Tris-HCl, 1 mM $MgCl_2$ at pH 7.5. Melting curves were obtained in the reverse and forward melting directions in a quartz cuvette of 1-cm path length with a temperature gradient of 20° to 90°C and a ramping rate of 1°C per min by monitoring the change in UV absorbance at 260 nm at each temperature (n=2).

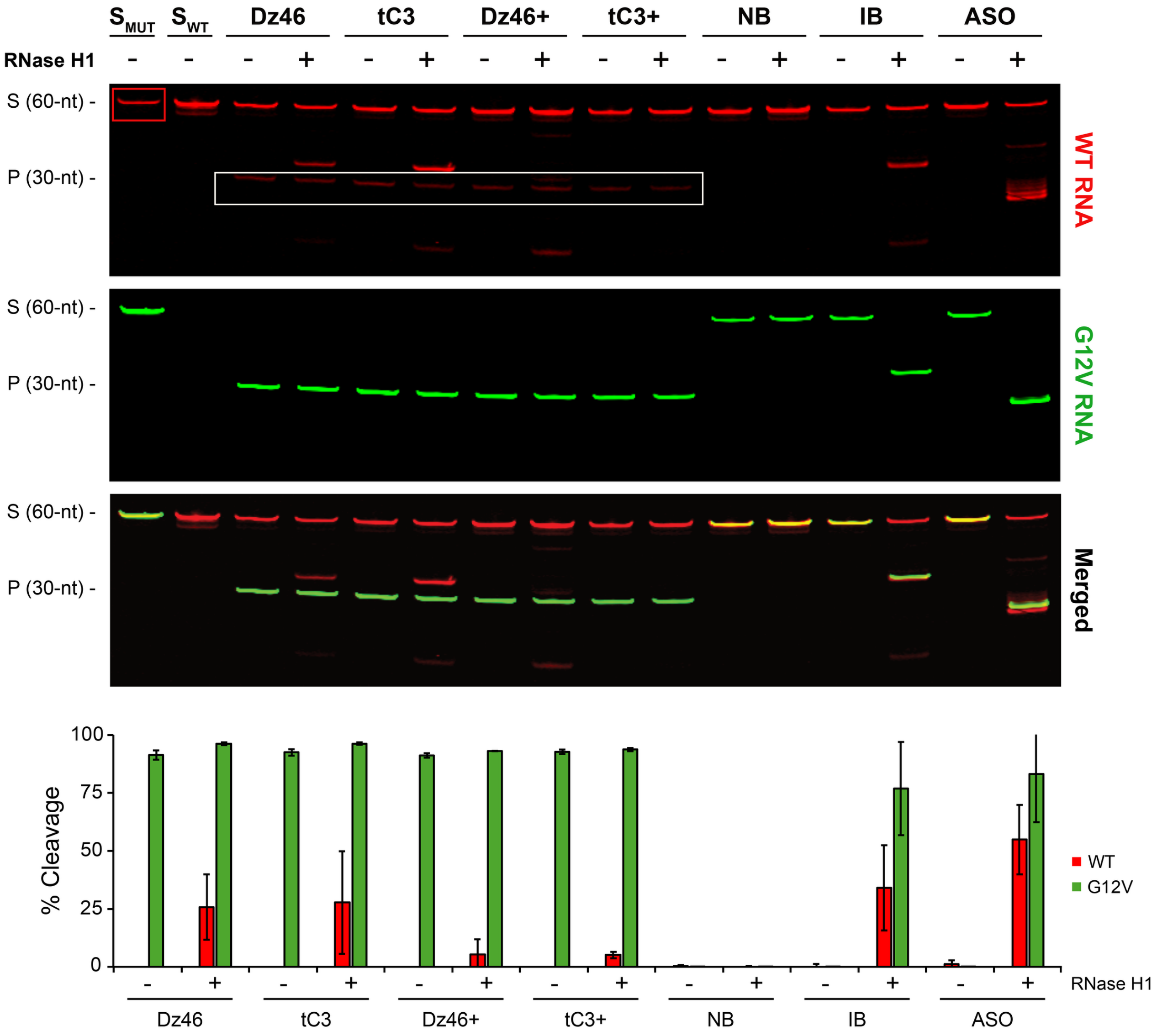

Supplementary Figure 8. RNase H activity of final chosen Dz46 variants carrying TNA modifications in the catalytic core and binding arms. Representative denaturing PAGE gel showing RNA cleavage profiles in reactions containing both the wild-type (red) and G12V (green) substrates (1:1) in the absence (-) or presence (+) of 5 ng/μL human RNase H1 after a 30 min incubation (n=2). Individual Cy5 (red) and AlexaFluor750 (green) channels, as well as the merged image, are shown. The corresponding bar graph shows the percent cleavage of WT (red) and G12V (green) substrate. The red band at P30 was excluded from the analysis due to signal bleed through in the 700 nm channel (white box). Reactions performed under simulated physiological conditions in buffer containing 1 mM $MgCl_2$, 50 mM Tris (pH 7.5), 10 mM NaCl, and 140 mM KCl at 37°C. S: 5'-labeled full-length substrate, P: 5'-labeled cleavage product.

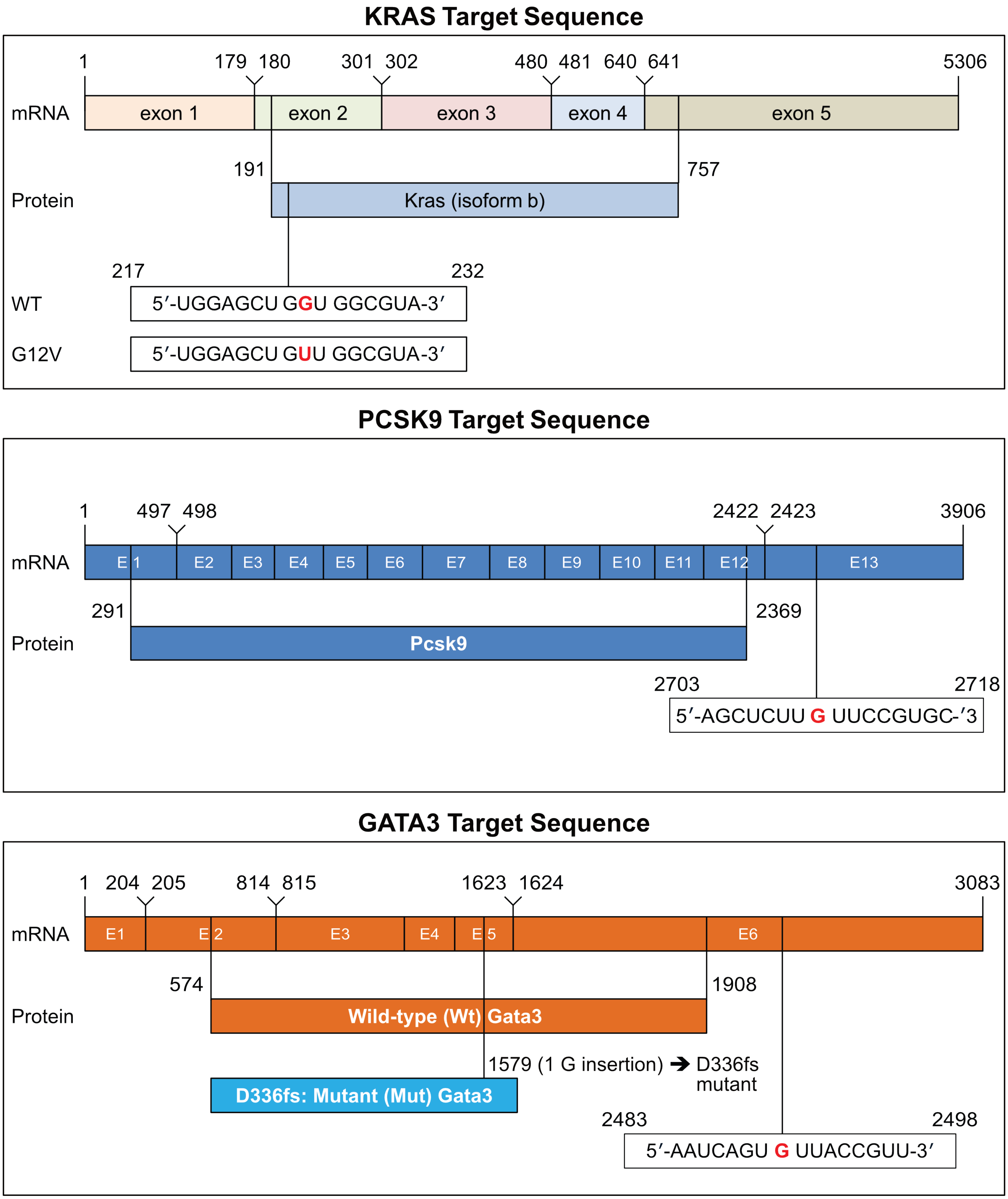

Supplementary Figure 9. Protein targets chosen for knockdown assays. WT KRAS mRNA sequence was obtained from NCBI (Accession number: NM_004985). Target sequence of G12V mutant derived from a single point mutation (G→U) is shown along with the WT. Human PCSK9 mRNA sequence was obtained from NCBI (Accession number: NM_174936.4). Human GATA3 mRNA sequence was obtained from NCBI (Accession number: NM001002295.2). GATA3 targeting Dz does not discriminate WT and mutant.
